# Small Molecule Mediated Restoration of Mitochondrial Function Augments Anti-Mycobacterial Activity of Human Macrophages Subjected to Cholesterol Induced Asymptomatic Dyslipidemia

**DOI:** 10.3389/fcimb.2017.00439

**Published:** 2017-10-10

**Authors:** Suman Asalla, Krishnaveni Mohareer, Sharmistha Banerjee

**Affiliations:** Molecular Pathogenesis Laboratory, Department of Biochemistry, School of Life Sciences, University of Hyderabad, Hyderabad, India

**Keywords:** dyslipidemia, *Mycobacterium tuberculosis*, mitochondria, small molecule M1, pre-disease, cholesterol, infection

## Abstract

*Mycobacterium tuberculosis* (*M.tb)* infection manifests into tuberculosis (TB) in a small fraction of the infected population that comprises the TB susceptible group. Identifying the factors potentiating susceptibility to TB persistence is one of the prime agenda of TB control programs. Recently, WHO recognized diabetes as a risk factor for TB disease progression. The closely related pathological state of metabolic imbalance, dyslipidemia, is yet another emerging risk factor involving deregulation in host immune responses. While high cholesterol levels are clinically proven condition for perturbations in cardiac health, a significant fraction of population these days suffer from borderline risk cholesterol profiles. This apparently healthy population is susceptible to various health risks placing them in the “pre-disease” range. Our study focuses on determining the role of such asymptomatic dyslipidemia as a potential risk factor for susceptibility to TB persistence. Macrophages exposed to sub-pathological levels of cholesterol for chronic period, besides impaired release of TNF-α, could not clear intracellular pathogenic mycobacteria effectively as compared to the unexposed cells. These cells also allowed persistence of opportunistic mycobacterial infection by *M. avium* and *M. bovis* BCG, indicating highly compromised immune response. The cholesterol-treated macrophages developed a foamy phenotype with a significant increase in intracellular lipid-bodies prior to *M.tb* infection, potentially contributing to pre-disease state for tuberculosis infection. The foamy phenotype, known to support *M.tb* infection, increased several fold upon infection in these cells. Additionally, mitochondrial morphology and function were perturbed, more so during infection in cholesterol treated cells. Pharmacological supplementation with small molecule M1 that restored mitochondrial structural and functional integrity limited *M.tb* survival more effectively in cholesterol exposed macrophages. Mechanistically, M1 molecule promoted clearance of mycobacteria by reducing total cellular lipid content and restoring mitochondrial morphology and function to its steady state. We further supported our observations by infection assays in PBMC-derived macrophages from clinically healthy volunteers with borderline risk cholesterol profiles. With these observations, we propose that prolonged exposure to sub-pathological cholesterol can lead to asymptomatic susceptibility to *M.tb* persistence. Use of small molecules like M1 sets yet another strategy for host-directed therapy where re-functioning of mitochondria in cholesterol abused macrophages can improve *M.tb* clearance.

## Introduction

Tuberculosis (TB), caused by the bacillus *Mycobacterium tuberculosis* (*M.tb*), affects one-third of the global population, despite being completely curable with an unfortunate exception of totally resistant *M.tb* (World Health Statistics, 2017). Surprisingly, <10% of immunocompetent people infected with *M.tb* develop the disease, while an astounding 90% successfully control the infection without showing any disease symptom, suggesting that only a minor fraction constitute the susceptible group (ATS, 2000). While issues like emergence of drug resistance, inability to identify latent cases and co-epidemic with HIV cloud effective TB management, yet another pressing need is to identify factors that are responsible for causing susceptibility to TB. Genetic susceptibility to TB has been long established in animal models demonstrating that genetic resistance or susceptibility to an infection can be bred into a population (Hoal, 2002). While malnutrition was always linked to TB susceptibility (Lonnroth et al., 2010; Cegielski et al., 2012), of late, lifestyle factors leading to dysglycemia, dyslipidemia, or even change in gut microbiota due to aberrant antibiotic use have been linked to *M.tb* susceptibility and survival (Khan et al., 2016). What seems to be of utmost importance is the innate immune response at the time of infection which decides if the bacteria will be eliminated or will survive in its niche, primarily alveolar macrophages. Clinical screenings have often identified conditions that range from health and disease, classifying the stages as “pre-disease.” These pre-disease states, if can be intervened effectively may reduce the susceptibility, persistence and progression of TB infection to TB disease.

Diabetes and obesity have been associated with TB disease progression (Hanrahan et al., 2010; Kumar and Babu, 2017). However, the role of closely related pathological state of metabolic imbalance, dyslipidemia, in host immune response to infections has not been addressed adequately. Dyslipidemia, manifested by high levels of total cholesterol, is either cause or consequence of several pathological conditions, like Type 2 diabetes mellitus (T2DM), excessive alcohol consumption, liver diseases and nephrotic syndrome (Goldberg, 2001; Kronenberg, 2005; Katsiki et al., 2016). There are also rising clinical evidence that suggest that chronic levels of “borderline” high cholesterol can dramatically increase the risk of cholesterol associated complications by nearly 40% later in life (Nelson, 2013). Besides, TB is also known to cause both hyperglycemia and hypercholesterolemia in patients (Padmapriyadarsini et al., 2011). Recent reports have indicated that pathogen-induced dysregulation of host lipid synthesis and sequestering in macrophages leads to cholesterol-loaded macrophages, called “foamy macrophages” which are critical components in both bacterial survival and dissemination (Russell et al., 2009). These macrophages are characterized by increased total cellular lipids constituting cholesterol and triacylglycerols (TAGs). In other studies, hypercholesterolemia has been shown to cause the death of pancreatic β-cells thereby promoting diabetic like condition (Hao et al., 2007). Using a mouse model the relationship between hypercholesterolemia, mitochondrial damage and cardiovascular-tissue function was determined, where they concluded that cardiovascular disease risk factors- hypercholesterolemia and second hand smoke cause mitochondrial damage and dysfunction (Knight-Lozano et al., 2002). Exogenous treatment of cholesterol induced pancreatic β-cell apoptosis through oxidative stress (Lu et al., 2011). Accumulation of cholesterol in mitochondrial membrane interferes with oxidative phosphorylation and anti-oxidant defense that result in the generation of ROS which culminates in cell death (Bosch et al., 2011). Further, it was shown that cholesterol induced damage in pancreatic β-cells is channelized through mitochondrial dysfunction (Asalla et al., 2016). These studies clearly indicate that hypercholesterolemia can lead to mitochondrial dysfunction that can get manifested in various pathological conditions.

Mitochondrial dysfunction, besides impaired phagosome maturation and lysosomal storage disorders, is decisive in the outcome of macrophage infection by pathogenic mycobacteria (Duan et al., 2002; Berg et al., 2016). Mitochondrial damage disturbs the balance of pro- and anti-apoptotic factors during *M.tb* infection, orienting the cell-death pathways to necrosis instead of apoptosis and helping mycobacterial dissemination. Specific *M.tb* factors like Rv2626c and Rv3873 (PPE68) have been implicated in promoting necrosis and dissemination (Danelishvili et al., 2016) while Rv2456c inhibited apoptosis (Jurcic Smith and Lee, 2016). Virulent mycobacteria, but not avirulent mycobacteria, have also been shown to induce significant variations to mitochondrial transmembrane potential, promoting necrosis (Chen et al., 2006). With these independent linkages between cholesterol, mitochondrial damage and mycobacterial pathogenesis, we speculated if cholesterol induced mitochondrial damage can lead to susceptibility to mycobacterial persistence.

While cardiac problems are apparently the most studied consequence of hypercholesterolemia, the metabolic linkages between cholesterol, diabetes and immune functions render a more complex situation. With these complex inter-relationships culminating into metabolic disorders, we sought to determine the role of asymptomatic dyslipidemia as a potential “pre-disease” condition and a risk factor for TB progression. Here we refer to the condition where individuals are clinically healthy but exhibit borderline risk cholesterol profile, that is, total cholesterol (TC): HDL ranging from 4.5 to 11 and LDL: HDL cholesterol ranging from 3 to 6 (Millan et al., 2009). Since such individuals are clinically healthy, these levels of cholesterol have been considered as sub-pathological. To address this, we established a cell culture based infection model where human macrophages were exposed to 80 μM cholesterol that retained cell viability throughout the experiment mimicking sub-pathological cholesterol exposure. Using this model, we showed that macrophages exposed to sub-pathological cholesterol levels for the chronic duration, remained viable, but were highly susceptible to not only pathogenic mycobacteria like H37Rv but also opportunistic mycobacteria like *M. avium* and *M. bovis* BCG. We further deciphered that this is mediated through mitochondrial dysfunction. We found that restoring these mitochondrial parameters to a steady state by pharmacological small molecule M1 reinstated the ability of intracellular mycobacterial clearance in cholesterol treated macrophages. We confirmed these inferences with PBMC derived macrophages from healthy volunteers showing borderline risk serum cholesterol profile. With this study, we propose that sub-pathological level of cholesterol is a “pre-disease” condition for TB persistence and small molecules like M1 hold the potential to be developed as a host-directed therapy for TB management.

## Materials and methods

### Bacterial cultures and infections

The pathogenic *M.tb* laboratory strain H37Rv, *M. bovis* BCG and *M. avium* (MTCC1723) were grown in Middlebrook 7H9 broth supplemented with 10% OADC (Himedia), 0.5% (v/v) glycerol and 0.05% (v/v) Tween-80. The culture was checked for possible contamination using Ziehl-Neelsen (ZN) staining procedure. Bacteria were grown in 7H9 broth with gentle shaking to an OD_600_ of 1.0 and harvested at 3,500 × g for 5 min. The cells were then resuspended in RPMI without antibiotics and dispersed by passage through a 100 U insulin syringe 10 times. The cells were counted following McFarland Nephelometer standards. MOIs were calculated based on these numbers and used for infection for 4 h at 37°C with 5% CO_2_. The cells were washed 4 h post-infection thrice with PBS, followed by suspension in RPMI complete media with antibiotics and further incubated for the indicated time at 37°C with 5% CO_2_ and processed as per the experiments described. All the experiments involving mycobacterial strains were performed in BSL3 facility following appropriate biosafety protocols approved by Institutional Biosafety committee (IBSC No: SB-N-98).

### Cell culture and PBMC assays

The infection assays and CFU enumeration assays were performed using either human monocytic cell line, THP-1, or PBMCs isolated from healthy volunteers.

The human monocytic cell line, THP-1 (ATCC) was maintained in RPMI 1640 medium supplemented with 10% (v/v) FBS and differentiated using 25 ng/mL PMA overnight followed by a resting period of 10–12 h. Subsequently, cholesterol treatment of differentiated THP-1 cells was carried out following the method described by Lu et al. with slight modifications. 250X (~7 mM) Cholesterol Lipid Concentrate (CLC) (GIBCO, Grand Island, NY, USA) was diluted in RPMI and then added to THP-1 cells at indicated time points and concentrations. In the case of infection, cells were pre-treated with cholesterol (80 μM) for 12 h followed by infection for 4 h as described. Small molecule M1 (Sigma) treatment was given post-infection till the rest of the assay.

Peripheral Blood Mononuclear Cells (PBMCs) isolation was performed as per the protocol described by Panda et al. (2012). Fifteen milliliter of peripheral blood was taken from clinically healthy human volunteers (*N* = 7) after taking prior written consents from subjects as per approved guidelines of Institutional Ethics Committee (IEC). These volunteers were profiled for their total serum cholesterol (TC), HDL, and LDL cholesterol from an NABL^1^ (Govt. of India) accredited diagnostic lab. The PBMCs were isolated from heparinized blood by density gradient centrifugation method using Histopaque (Sigma). Briefly, the blood from each individual was diltuted twice with RPMI complete media and gently overlayed on Histopaque at 1:1 ratio followed by centrifugation at 100 × g for 30 min. The buffy coat layer containing PBMCs was gently aspirated and transferred into sterile centrifuge tubes. The cells were harvested and washed with RPMI and further cultured in complete RPMI media supplemented with antibiotics. The cell number was quantified and adjusted to 0.2 × 10^6^ cells/well in 24 well plates. The cells were cultured for 7 days at 37°C. The non-adherent cells were eliminated by washing with RPMI media and the adherent cells were used for infections.

The various experimental categories included (i) cholesterol untreated uninfected; (ii) cholesterol treated uninfected; (iii) Cholesterol untreated infected; (iv) cholesterol treated infected; (v) cholesterol untreated uninfected, M1 treatment (vi) cholesterol untreated infected, M1 treatment (vii) cholesterol treated uninfected, M1 treatment and (viii) cholesterol treated infected, M1 treatment.

### CFU enumeration of viable intracellular bacilli

For the CFU count, 0.2 × 10^6^ THP-1 cells were seeded per well in a 24-well plate and infected with mycobacteria as per the experiments described. At the end of the experiment, infected cells were washed five times with PBS to remove any residual extracellular bacilli. The cells were then lyzed with sterile water at 37°C for 10 min. The lysates were diluted and plated on 7H10 agar plates. The plates were incubated at 37°C with 5% CO_2_ and 95% humidity for 2–3 weeks and the colonies were enumerated.

Intracellular mycobacterial survival was also evaluated using Alamar Blue assays as described earlier (Ganji et al., 2016).

### MTT assay

MTT assay was performed as per Vemula et al. (2016). Briefly, at the end of the experiment, MTT dye (5 mg/mL) was added at 25 μL per 100 μL media for 2 h. The resulting formazan crystals were solubilized by 100 μL of DMSO. The absorbance was measured at 540 nm using a multimode reader (BioTek).

### Estimation of total cellular cholesterol content in macrophages

THP-1 cells were seeded in 6 well plates at a density of 2 × 10^6^ cells/well and cultured overnight in complete medium. Following cholesterol treatment for indicated time, the amount of total cellular cholesterol in macrophage cell lysate was measured using commercial enzyme-based cholesterol assay kit (Cat# STA-384 Cell Biolabs, Inc.) according to manufacturer's instructions.

### Oil Red O staining

Oil Red O staining was performed as per the protocol described in Ganji et al. (2016). The cells were washed with PBS, fixed with 10% formalin at room temperature for 30 min. The cells were then washed with sterile water and incubated with 60% isopropanol for 5 min, followed by staining with 60% Oil Red O Stain at 37°C for 5 min in the dark. The excess stain was washed thrice with sterile water. Intracellular lipid bodies were observed microscopically. The amount of lipid bodies were quantified by eluting the intracellular Oil Red O Stain using 100% isopropanol at room temperature for 10 min and measuring the intensity at 500 nm spectrophotometrically with 100% isopropanol as blank.

### Analysis of mitochondrial membrane potential (MMP) (ΔψM)

Mitochondrial membrane potential (MMP) was measured by JC-1 staining kit (Cat. no. CS0390, Sigma) following manufacturer's instructions. 0.2 × 10^6^ THP-1 cells were seeded in 24 well plates and cultured overnight in complete medium. At the end of the experiment, cells from the different experimental categories described earlier were assayed. Briefly, the cells were washed with PBS and stained with 2.5 μM of JC-1 in 1X JC-1 staining buffer. These cells were further incubated for 20 min at 37°C in a humidified atmosphere containing 5% CO_2._ Further, the cells were washed with ice-cold 1X JC-1 staining buffer. The cells were then resuspended in 0.5 ml of the same buffer and analysis of the samples was performed using spectrofluorimetry (spectramax m5). JC-1 monomers were detected in the FL1 channel (green) and the aggregates were detected in FL2 channel (red), and the ratio of FL2/FL1 was calculated for determination of mitochondrial membrane potential.

### Detection of ADP and ATP levels

ATP and ADP levels were measured in cells of different experimental categories as described earlier by using ADP/ATP Ratio Assay Kit (Bioluminescent, ab65313), based on the bioluminescent detection, following manufacturer's instructions.

### Analysis of mitochondrial ROS

THP-1 cells at a density of 0.2 × 10^6^ cells per well were seeded in 6 well plates and incubated overnight in complete medium. At the end of the experiment, cells from experimental categories, described in the text, were stained for 30 min with fluorogenic dye MitoSOX™ Red for mitochondrial ROS measurement following manufacturer's (Molecular Probes) instructions. Analysis of the samples was performed using spectrofluorimetry (spectramax m5).

### Confocal microscopy

2 × 10^6^ THP-1 cells were seeded in six-well plates containing 20-mm-diameter glass coverslips and cultured overnight. At the end of the experiment, cells from various experimental categories were washed with PBS and stained with 50 nM Mito-Tracker Red (M7512) in complete medium at 37°C for 30 min, washed again with PBS and finally fixed with 4% paraformaldehyde. The coverslips were then mounted in Vectashield (Vector Laboratories), and images were visualized in confocal laser scanning microscope (Leica, Germany) with 63 × oil immersion objective lens.

### Real-time PCR

Extraction of RNA from experimental cells was performed using trizol method as per manufacturer's instructions (Invitrogen). Two microgram of total RNA from each experimental category was used for reverse transcription using iScript cDNA Synthesis Kit as per manufacturer's protocol (BioRad) using 2X SYBR green mix (iTaq™ Universal SYBR Green Supermix, BioRad). GAPDH gene was used as endogenous control. The primers used for qRT-PCR are tabulated in Table S1. The real-time PCR data analyses were carried out using the 2^−ΔΔCt^ method (Schmittgen and Livak, 2008).

### Enzyme linked immunosorbent assay

2 × 10^6^ THP-1 cells were seeded in six-well plates and followed by cholesterol treatment and infection. Cell culture supernatant was collected and filtered through 0.22 μm syringe filter after infection and indicated time. For measurement of cytokine levels, BD OptEIA enzyme-linked immunosorbent assay (ELISA) kit was used following manufacturer's instructions. The absorbance was measured at 450 and 570 nm in multimode reader (BioTek). Each sample was assayed in triplicate.

### Transmission electron microscopy (TEM)

The protocol was essentially followed as reported previously (Rajapakse et al., 2001) with minor modifications. Briefly, cells from each experimental category were fixed in 2.5% glutaraldehyde in 0.1 M phosphate buffer (pH 7.4) for 24 h at 4°C and washed with PBS twice each for 45 min, then post-fixed in 1% aqueous Osmium Tetraoxide (OsO4) for 2 h. Subsequently, the cells were washed with deionized distilled water for four times each for 45 min, dehydrated in series of graded alcohols followed by infiltration and finally embedded in araldite 6005 resin or spur resin. The cells were then incubated at 80°C for 48 h for complete polymerization. Ultra thin (60 nm) sections were made with a glass knife on an ultramicrotome (Leica Ultra cut UCT-GA-D/E-1/00), followed by mounting on copper grids and stained with saturated aqueous Uranyl acetate (UA) and counterstained with Reynolds lead citrate (LC). Sections were viewed under TEM (JEOL from Japan). Images were acquired digitally.

### Statistical analysis

Each experiment was carried out at the least three times (or more). The data are presented as mean ± *SD* (standard deviation). The statistical significance was assessed by *t*-test for the data distributed normally as inferred by normality test, for other data Mann-Whitney test was applied. This was performed using SigmaPlot version 11.0. A *p*-value of ≤ 0.05 was considered statistically significant.

## Results

### Chronic exposure to sub-pathological levels of cholesterol impaired macrophages from clearing intracellular *mycobacterium tuberculosis* H37Rv

**Figure 1:**
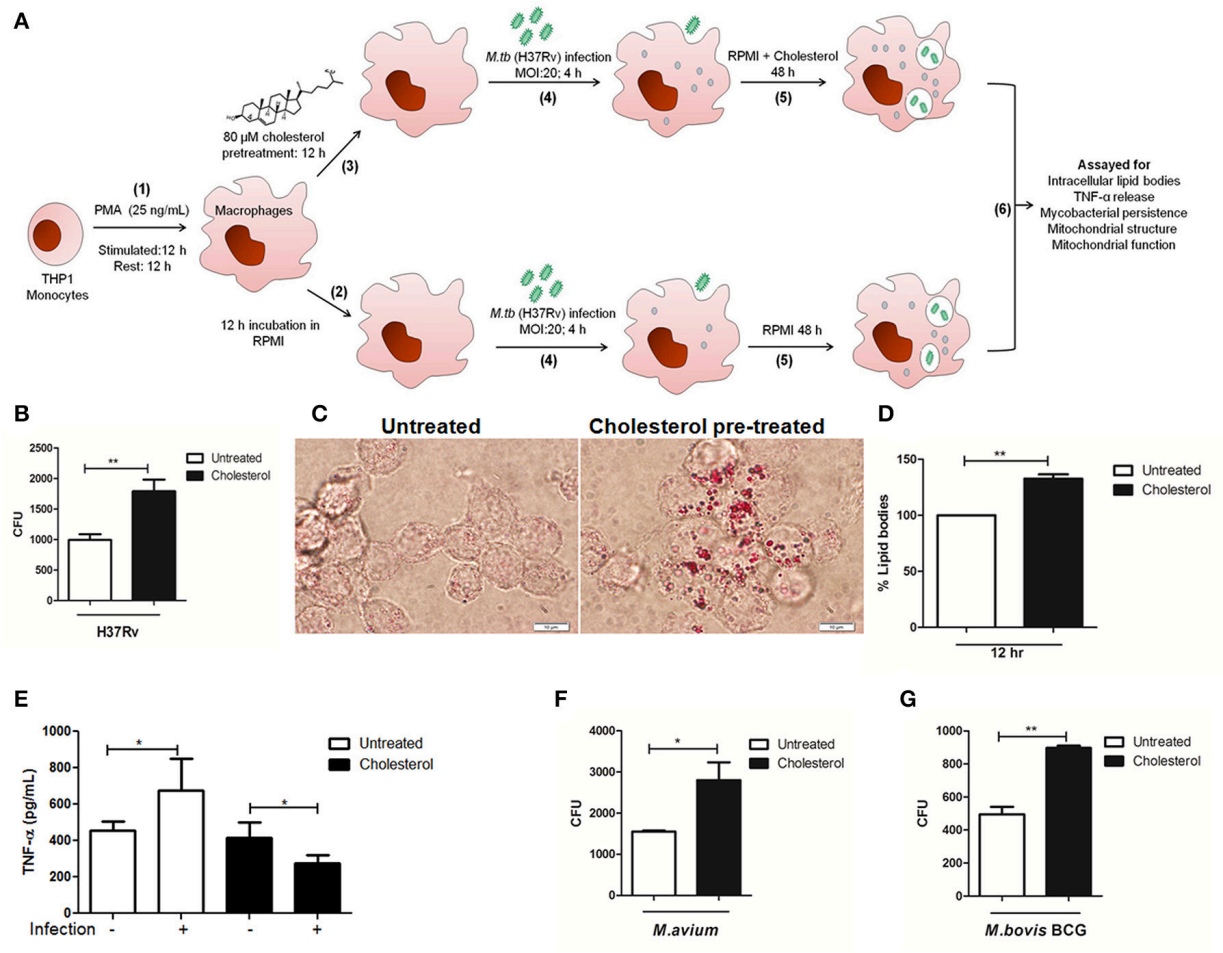
Chronic exposure of THP-1 cells to sub-pathological levels of cholesterol impaired them from clearing intracellular Mycobacteria. **(A)** Schematic representation of the cell culture based model used in the study **(B)** Measurement of the clearance of intracellular pathogenic mycobacteria H37Rv by CFU counts in untreated and treated cells. **(C)** Oil Red O staining of intracellular lipid bodies in untreated and treated cells 12 h post cholesterol (80 μM) treatment before infection. The bright field images for each category are representative of at the least five fields (scale bar 10 μm). **(D)** Lipid bodies as visualized in **(C)** were quantified on microtiter plate spectroscopically after Oil Red O staining. **(E)** TNF-α released by untreated and cholesterol treated, with (MOI 20) or without H37Rv infection as measured by ELISA. Clearance of **(F)** non-tuberculous mycobacteria *M. avium* or **(G)** attenuated mycobacteria *M. bovis* BCG by untreated or treated THP-1 cells as evaluated by CFU. Each experiment was carried out at the least three times. Statistical analyses were performed as mentioned in methods. Error bars represent ± *SD*. ^*^P ≤ 0.05; ^**^P < 0.005.

With an aim to investigate the sequence of molecular events underlying the impact of long-term exposure (chronic) to sub-pathological levels of cholesterol on the clearance of intracellular pathogen *M.tb* by human macrophages, we first developed a suitable model for mycobacterial infection wherein human THP-1 monocytes, differentiated into macrophages using PMA, were infected with pathogenic mycobacteria H37Rv during exposure to different levels of cholesterol for 64 h (Figure 1). A systematic titration of cholesterol concentration, cholesterol exposure time and MOI of *M.tb* infection was exercised ensuring THP-1 remained viable during the course of experiments. The cells were treated with increasing concentrations of cholesterol (i.e., 40, 80, 160, and 250 μM cholesterol) for 24 h and evaluated for viability by using tetrazolium salt (MTT) metabolite assay. We observed that up to 80 μM of cholesterol, there was no effect on the viability of THP-1 while cholesterol concentration above 160 μM showed significant cell death (see Figure S1A). It was further observed that cells remained viable up to 72 h upon 80 μM cholesterol treatment, while 100 μM cholesterol treatment decreased the cell viability significantly (see Figure S1B). With these experiments, we concluded that 80 μM of cholesterol was the maximum concentration of cholesterol that could be tolerated by THP-1 cells for 48 h post-infection in our experimental conditions. Hence, treatment with 80 μM of cholesterol was subsequently used to represent borderline risk cholesterol (from now on referred to as sub-pathological cholesterol). After precise titrations of H37Rv infections, we observed that cholesterol treated THP-1 cells could withstand H37Rv infection at MOI 20 for 48 h (see Figure S1C), which was used for further studies. In summary, in our model, THP-1 cells were pre-treated with 80 μM cholesterol for 12 h followed by mycobacterial infection (MOI 20) for 4 h and were kept in sustained presence of 80 μM cholesterol for 48 h post-infection. The total exposure time of cholesterol in our experimental set-up was 64 h, after which assays were carried out, (Figure 1A). Since cells remained viable throughout the experimental period, these simulated conditions where cells exposed to “borderline risk cholesterol” were “asymptomatic and apparently healthy”.

Using this model, the efficacy of these macrophages to eliminate intracellular H37Rv was then evaluated. THP-1 cells were infected with H37Rv in the presence and absence of cholesterol and live intracellular H37Rv were scored through CFU. We observed significantly high CFU counts from THP-1 treated with cholesterol, showing 1790 ± 192.0 CFU as against 991.7 ± 94.35 CFU seen from untreated THP-1 (Figure 1B). Thus, there was an increase in live persistent bacteria by 80.5% in cholesterol-treated cells. Since some of the earlier cell culture-based studies have implicated the role of cholesterol in supporting entry of intracellular pathogens like *Brucella* and *M. bovis* into macrophages (Gatfield and Pieters, 2000; Naroeni and Porte, 2002; Viswanathan et al., 2015), we monitored mycobacterial CFU in treated and untreated THP-1 cells at 0 h post-infection (see Figure S1D). The results showed that there was an insignificant difference in mycobacterial CFU at 0 h in both treated (1,788 ± 411.5 CFU) and untreated cells (1,705 ± 271CFU) clearly showing that cholesterol concentration and exposure time in our model did not affect mycobacterial entry. This suggested that intracellular events post-mycobacterial entry may have supported mycobacterial survival in the cholesterol exposed THP-1 cells.

Having observed that 64 h of sub-pathological cholesterol exposure impaired clearance of pathogenic mycobacteria by otherwise viable macrophages (Figure 1B), we checked the levels of intracellular cholesterol and lipid bodies in these cells. Recent studies have pointed that, macrophages with accumulated lipid bodies (foamy macrophages) contribute to tissue pathology and exhibit limited protection against persistent bacteria (Russell et al., 2009; Daniel et al., 2011). At the same time, pathogen-induced dysregulation of host lipid synthesis and requisitioning have been shown to impair protective immunity against TB in a mouse model (Martens et al., 2008). While such observations have been made with high cholesterol treatments, we observe that even chronic exposure to sub-pathological cholesterol levels resulted in the accumulation of cholesterol in macrophages. Intracellular lipid and total cellular cholesterol concentrations were evaluated as described in methods. Both microscopic examination (Figure 1C) and spectrophotometric quantification (Figure 1D) showed that cholesterol pre-treatment for 12 h in THP-1 cells resulted in an increase of intracellular lipid bodies by 32.7% and 90.5% when sustained for next 48 h (please refer **Figure 3B**), giving these cells a sustained “foamy” phenotype during the course of the experiment.

We then investigated the potential of these cells to release TNF-α, a potent pyrogenic cytokine that stimulates the acute phase of the immune response and is also one of the first cytokines to be released in response to a pathogen (Beutler, 1999). TNF-α plays a key role both in initial and chronic phase of *M.tb* infection (Lin et al., 2007). As reported earlier, we observed an induction of TNF-α secretion in response to H37Rv infection (1.48-fold increase as compared to unifected, untreated). However, it was observed that both treated and untreated THP-1 cells released a similar amount of TNF-α (Treated 417.7 ± 37.17 and untreated 454.5 ± 22.92 pg/mL). But upon H37Rv infection, the treated cells failed to release adequate levels of TNF-α (276.5 ± 19.94 pg/mL) as compared to untreated infected cells (675.6 ± 87.86 pg/mL) (Figure 1E). Taken together, these experiments showed that while cholesterol exposed cells were viable, they failed to respond appropriately to mycobacterial infection.

Having observed a compromised protective response by cholesterol treated infected cells, we hypothesized that such microenvironment could make macrophages susceptible to persistent infections even by opportunistic mycobacteria. To test the same, we infected treated and untreated THP-1 cells with non-tuberculous pathogenic mycobacteria (NTM) *M. avium* and attenuated mycobacteria, *M. bovis* BCG. We observed that as compared to untreated (1,550 ± 28.87 CFU), there was an increase of 80.64% of persistent *M. avium* (2,800 ± 435.9 CFU) in cholesterol treated THP-1 cells (Figure 1F), while for *M. bovis* BCG it was observed to be increased by 81.2% (untreated 495.0 ± 45.37 CFU and treated 897.0 ± 13 CFU) (Figure 1G). The increase in persistent bacteria inside cholesterol treated THP-1 cells indicated that these cells are relatively immune-compromised and provide a conducive environment to these opportunistic mycobacteria for their growth and survival. Collectively, with these experiments, we conclude chronic exposure of macrophages to sub-pathological cholesterol creates a conducive environment in the form of foamy phenotype, setting a “pre-disease” state in the cells supporting *M.tb* persistence. We further investigated the molecular events underlying the same using this model.

### Chronic exposure to sub-pathological cholesterol impaired mitochondrial function in infected macrophages which could be rescued using small molecule M1

Some of the earlier assays with exogenous treatment of cholesterol suggested mitochondrial dysfunction resulting in apoptotic cell death (Lu et al., 2011). While most of these studies advocated that it leads to activation of apoptosis, our model clearly has taken conditions where cell death is minimal upon exogenous treatment of cholesterol.

**Figure 2:**
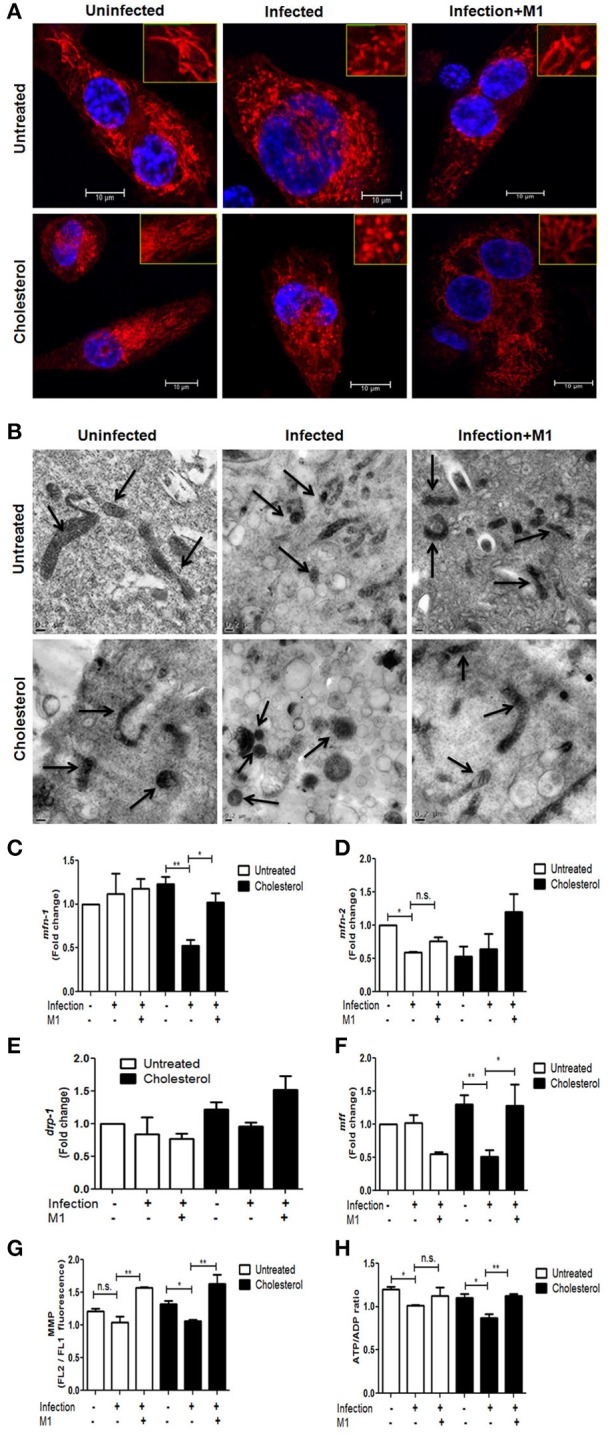
Mycobacterial infection in cholesterol treated THP-1 cells altered mitochondrial structure and function, which was rescued by small-molecule M1. **(A)** Confocal microscopy showing distribution and shape of mitochondria stained by mito-tracker red (seen in red fluorescence) against a background of DAPI stained cells from each category. The images are representative of at the least five fields (scale bar 10 μm). The inset in each image is the zoomed image showing the gross mitochondrial shape. **(B)** Transmission electron micrographs depicting changes in the mitochondrial morphology from cells from each category. Arrows indicate representative mitochondria. The bar represents 0.2 μm scale. qRT-PCR-based changes in mRNA expression levels of mitochondrial fusion genes *mfn-1* **(C)** and *mfn-2* **(D)** as well as mitochondrial fusion genes *drp-1* **(E)** and *mff* **(F)** in each category. Fold change in transcript levels were calculated as against untreated and uninfected THP-1 cells. The transcript levels were quantified by relative 2^−ΔΔCt^ normalized against endogenous GAPDH. **(G)** Measurement of Mitochondrial Membrane Potential in each category as described in methods. The data is expressed as the ratio of FL2 to FL1. **(H)** Graphical representation of intracellular ATP/ADP ratios in each category using bioluminescence assay as described in methods. Each experiment was carried out at the least three times. Statistical analyses were performed as mentioned in methods. Error bars represent ± SD. ^*^P ≤ 0.05; ^**^P < 0.005.

To check if sub-pathological cholesterol can increase the risk of mitochondrial malfunction upon infection, we first studied alterations in mitochondrial structure microscopically. For this, mitochondrial morphology was monitored using fluorescent staining and transmission electron microscopy (TEM). Fluorescent staining of mitochondria from untreated and treated THP-1 cells, with or without infection, using MitotrackerRed showed distinct morphological differences (Figure 2A). In the control cells, the majority of mitochondria were observed to be rod-shaped, elongated, and forming interconnected structures, while infected cells showed predominantly nod-shaped, ovoid and solitary mitochondria, indicating stress morphology (Figure 2A, upper panel, columns i and ii). Such stress morphology could be observed in cholesterol exposed THP-1 cells even before infection, which augmented several fold upon infection where solitary spheres and rods of various sizes were predominantly seen (Figure 2A, lower panel, column i and ii). These observations were also supported by TEM imaging (Figure 2B). Transmission electron micrographs clearly show that mitochondrial morphology in control cells was predominantly elongated rod-shaped and interlinked, while the same in either cholesterol treated or infected cells were predominantly ovoid nod-shaped and solitary (Figure 2B, lower panel column i and upper panel column ii, respectively). Infection of cholesterol treated THP-1 cells resulted in a more noticeable change in mitochondrial morphology where mitochondria appeared swollen and spherical (Figure 2B, lower panel column ii). Additionally, we also looked for associated changes in nuclear morphology using confocal microscopy, where the images were focused on nuclei (Figure S5). We did not observe any changes in nuclear morphology. With these visual evidences, we could conclude that exposure to sub-pathological levels of cholesterol leads to changes in mitochondrial morphology, very similar to that caused by *M.tb* infection alone. This indicated that cholesterol exposed cells are already under stress, which gets several fold amplified upon infection as evident from swollen mitochondrial morphology.

To assess the mitochondrial structure in relation to changes in the expression of genes involved in mitochondrial dynamics, mRNA levels of mitochondrial fusion and fission genes *mfn-1, mfn-2, drp-1*, and *mff* in these macrophages were measured through qRT-PCR (Figures 2C–F). The levels of *mfn-1* and *mfn-2* were significantly decreased by ~2-fold in cholesterol treated infected cells, whereas *mfn-2*, but not *mfn-1*, was downregulated by ~2-fold during infection in untreated cells (Figures 2C,D). The levels of *drp-1* did not change significantly in any condition (Figure 2E). However, levels of *mff* were observed to be significantly reduced by ~2-fold in cholesterol treated infected cells as compared to cholesterol treatment alone (Figure 2F). Overall, the changes in the expression levels of mitochondrial fission and fusion genes support the variations in the mitochondrial structural dynamics as observed microscopically.

With the clear suggestion of changes in mitochondrial structure, we further studied the changes in mitochondrial function. MMP in untreated and cholesterol treated THP-1 cells, with and without infections were measured using fluorometric assays (Figure 2G). It was observed that while either infection or cholesterol alone did not significantly affect MMP, infection of cholesterol-treated cells caused a consistent (20.07%) reduction in MMP. Since the change in MMP should reflect in ATP production, in addition to this, mitochondrial function was assayed by measuring ATP/ADP ratio. ATP/ADP ratio in untreated infected cells was 15.65%, while that in treated infected cells was 21.08%, suggesting cumulative damage to mitochondria upon cholesterol treatment and infection together (Figure 2H). With these experiments, we registered a consistent change in mitochondrial functional parameters. Such subtle and consistent changes in mitochondria have been reported to have huge physiological consequences (Buckman and Reynolds, 2001) and should not be considered inconsequential. MitoROS production, upon infection of both untreated and treated cells, however, remained unaltered (please see Figure S2A). Further, as control experiments, to check if cholesterol concentrations <80 μM could cause alterations in mitochondrial structure and function, 20 μM and 40 μM cholesterol treatments were used to assay for their impact on both mitochondrial structure and function (Figures S2B,C) and clearance of mycobacteria (Figure S2D). We do not observe any significant change in mitochondrial morphology or function, neither any differences in mycobacterial clearance upon treatment with 20 and 40 μM cholesterol as compared to the untreated.

These results implicated that, prolonged exposure to sub-pathological cholesterol levels created an amicable environment for *M.tb* in otherwise apparently healthy macrophages, by compromising mitochondrial structure and function, which significantly decreases the anti-mycobacterial activity of infected cells, thereby promoting mycobacterial persistence.

We next attempted to restore the mitochondrial activity using a commercially available, pharmacological small molecule, M1, which has been reported to restore mitochondrial health in various systems upon different stresses (Wang et al., 2012). We standardized the effective concentration of M1 in our experimental conditions, keeping the cells viable (please see Figure S3A). THP-1 cells, untreated and cholesterol treated, with and without H37Rv infections, were treated with 30 μM of M1 post-infection for 48 h. All the mitochondrial parameters, viz; morphology, expression of genes responsible for mitochondrial dynamics, MMP and ATP/ADP ratio were reanalyzed in the presence of M1. We observed that mitochondrial morphology was substantially restored upon M1 treatment of both untreated and treated infected cells (Figure 2A lower panel column iii and Figure 2B lower panel column iii, respectively). M1 also restored *mfn1*and *mfn2* levels in cholesterol treated infected cells, but not in untreated infected cells (Figures 2C,D respectively). We, for the first time, observed that M1 restored levels of the fission gene *mff* in cholesterol treated infected cells (Figure 2F). The restoration of the expression levels of these genes, supported by re-establishment of morphological features, suggested that M1 probably reinstated the balance between fission and fusion events, and this equilibrium maintained the steady state mitochondrial dynamics, more efficiently in cholesterol-treated cells. We also observed that consequent restoration of MMP (Figure 2G) and ATP/ADP ratio (Figure 2H) by M1 during infection of cholesterol exposed cells.

### Rescuing mitochondrial function and reduction of intracellular lipid bodies by M1 increased mycobacterial clearance in cholesterol treated infected macrophages

**Figure 3:**
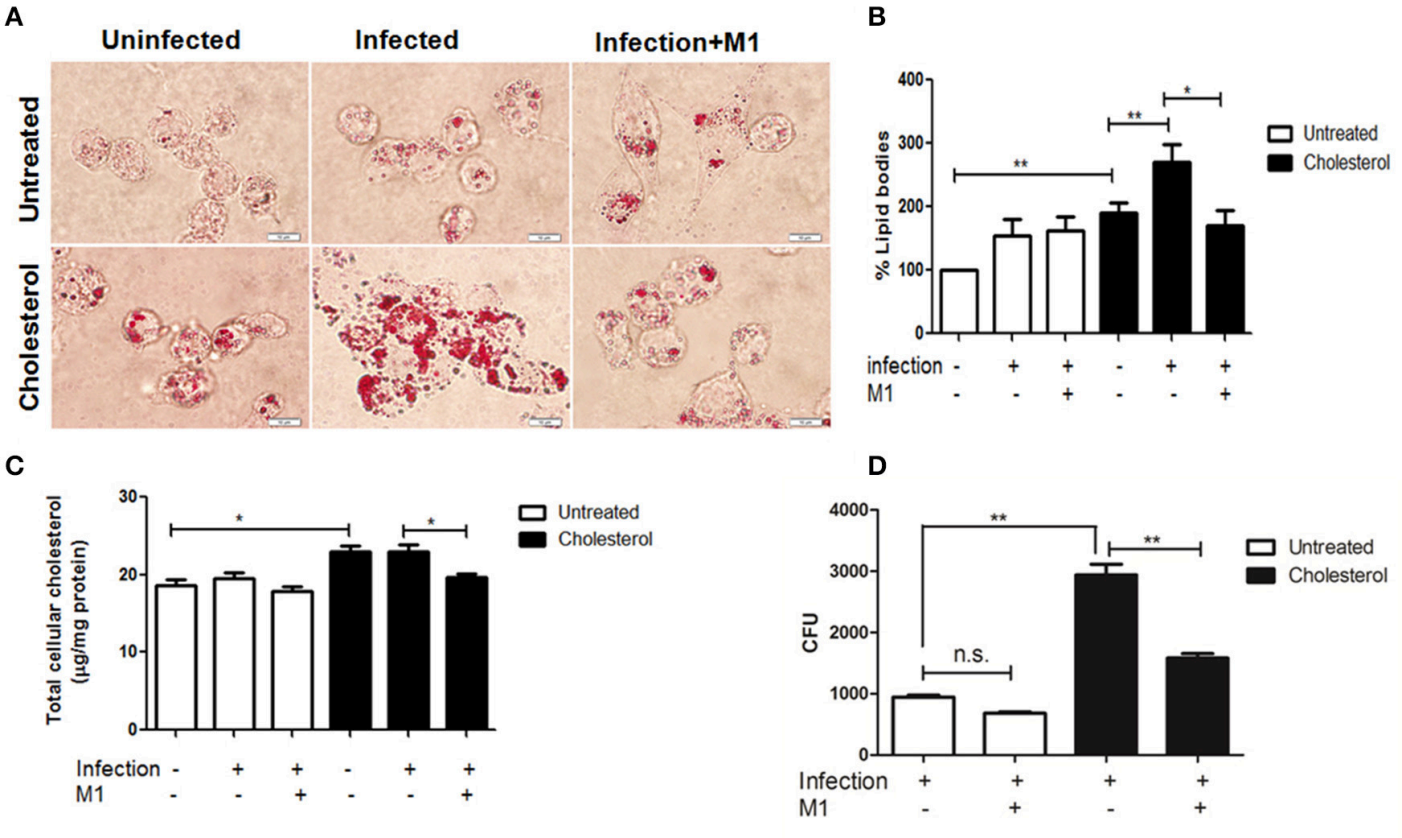
Pharmacological intervention of M1 improves mycobacterial clearance by cholesterol treated THP-1 cells. **(A)** Representative bright field microscopy image showing the accumulation of lipid bodies by Oil Red O staining inside the untreated or cholesterol treated H37Rv infected cells upon 48 h post-infection, with or without M1 treatment. The white bar indicates 10 μm scale. Images were taken at 100 × magnification. **(B)** Bar graph representing the fold change in the accumulation of Oil Red O Stain as measured at Abs500 nm in different categories. Percentage changes were calculated with respect to the values of the untreated uninfected THP-1 cell. **(C)** Colorimetric assay quantifying total cellular cholesterol in each category. **(D)** Measurement of H37Rv clearance by CFU counts upon M1 treatment of untreated and cholesterol treated infected THP-1 as described. Each experiment was carried out at the least three times. Statistical analyses were performed as mentioned in methods. Error bars represent ± SD. ^*^P ≤ 0.05; ^**^P < 0.005.

We next postulated if restoring mitochondrial structure and function in cholesterol exposed THP-1 by M1 will potentiate these cells for mycobacterial clearance. Having noted that M1 treatment rescued mitochondrial function in cholesterol treated THP-1, we checked the lipid bodies and intracellular cholesterol levels in these cells. In compliance with other studies, we observed that H37Rv infection alone resulted in the increase in lipid bodies by 53.77% (Figure 3A upper panel column ii and Figure 3B). This impact was more pronounced in cholesterol treated H37Rv infected cells which showed an increase in intracellular lipids by 76.2% as compared to infection in untreated cells (Figure 3A lower panel column ii and Figure 3B).

Besides restoring mitochondrial structure and function, when cholesterol exposed infected cells were treated by M1, it resulted in a decrease in the intracellular lipid bodies by 58% (Figure 3A lower panel column ii and iii and Figure 3B), as well as total intracellular cholesterol levels by 15% (Figure 3C). M1 treatment could not reduce either lipid bodies or cholesterol significantly in untreated infected cells (Figure 3A upper panel column ii and iii, Figures 3B,C). Finally, the impact of M1 on mycobacterial clearance was scored. Pharmacological intervention by M1 reduced the intracellular H37Rv burden of cholesterol treated infected cells by 46.16% and of untreated infected cells by 27.87%. This indicated that the efficacy of M1 in clearance of mycobacterial infection was higher in cholesterol treated macrophages (Figure 3D). It is to be noted that M1 treatment of cholesterol untreated and treated cells in the absence of infection had insignificant impact on intracellular lipid and MMP (Figures S3B–D). It is also to be noted that M1 had no impact on the *in-vitro* growth of H37Rv cultures (please see Figure S4).

### PBMC derived macrophages from subjects with borderline risk cholesterol profile showed reduced clearance of intracellular mycobacteria

**Figure 4:**
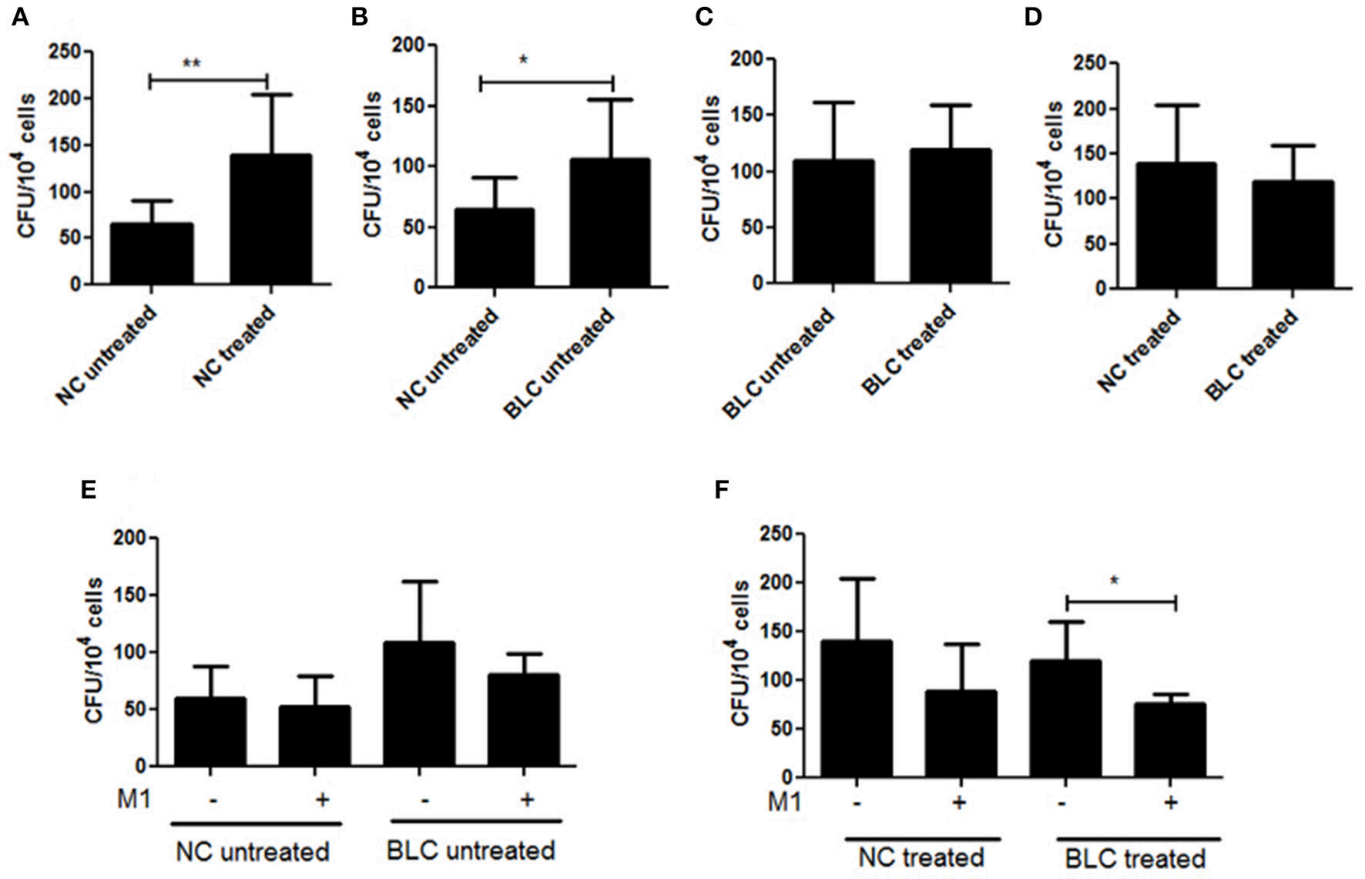
PBMC derived macrophages from subjects with borderline risk cholesterol profiles showed reduced clearance of intracellular mycobacteria. PBMC derived macrophages were isolated from human volunteers with either normal (NC) or borderline risk (BLC) cholesterol profiles, were either untreated or cholesterol treated and then infected with *M.tb* H37Rv as discussed in methods. The clearance of mycobacteria by PBMC derived macrophages from the two categories was evaluated by CFU enumeration 48 h post-infection. Bar plots showing CFU enumeration from **(A)** Cholesterol untreated and treated PBMC derived macrophages of NC; **(B)** Cholesterol untreated PBMC derived macrophages of NC and BLC; **(C)** Cholesterol Untreated and treated PBMC derived macrophages of BLC; **(D)** Cholesterol treated PBMC derived macrophages of NC and BLC; **(E)** Cholesterol untreated PBMC derived macrophages of NC and BLC with or without M1 treatment and **(F)** Cholesterol treated PBMC derived macrophages of NC and BLC with or without M1 treatment. Each experiment was carried out at the least three times NC (*n* = 4) and BLC (*n* = 3). Statistical analyses were performed as mentioned in methods. Error bars represent ± *SD*. ^*^P ≤ 0.05; ^**^P < 0.005.

From the above experiments, we could infer that sub-pathological long term exposure to cholesterol impairs macrophages from adequately responding to mycobacterial infection and prevents its clearance. The small molecule M1 could rescue cholesterol exposed infected macrophages and helped in improving mycobacterial clearance. To validate these cell line based observations in primary cells, we used PBMC derived macrophages from human volunteers with either normal (referred to as NC) or borderline risk (referred to as BLC) cholesterol profiles (Table S2). Mycobacterial clearance was assayed by CFU enumeration from PBMC derived macrophages subjected to different conditions as in the chronic cell culture model (Figure 4). Similar to observations from the cell culture model, we found that intracellular clearance of mycobacteria by cholesterol treated PBMC derived macrophages from NC (139.8 ± 22.84) was significantly reduced (46.3%) as compared to untreated cells (64.76 ± 9.29) (Figure 4A). We then compared the clearance of mycobacteria by PBMC derived macrophages from NC and BLC. As expected, PBMC derived macrophages from BLC (108.7 ± 20.21) showed reduced clearance (61.3%) of mycobacteria as compared to those from NC (64.76 ± 9.29) (Figure 4B). Further, there was no difference in clearance of mycobacteria from PBMC derived macrophages of BLC either cholesterol treated (119.9 ± 17.84) or untreated (108.7 ± 20.21) (Figure 4C). Also, there was no significant difference in the clearance of mycobacteria from cholesterol treated PBMC derived macrophages from either NC (139.8 ± 22.84) or BLC (119.9 ± 17.84) (Figure 4D). These experiments showed that exogenously treated PBMC derived macrophages from NC behaved similar to those of untreated BLC confirming that PBMC derived macrophages from BLC had reduced efficiency of mycobacterial clearance.

M1 treatment did not improve the clearance of mycobacteria significantly from PBMC derived macrophages from either NC or BLC. However, when we compared improvement of clearance of mycobacteria between untreated NC and BLC, we found that while M1 could improve clearance in NC by 12.9%, the same in BLC was 25.6% (Figure 4E). On the other hand, impact of M1 on mycobacterial clearance was more efficient in cholesterol treated PBMC derived macrophages from both NC and BLC (Figure 4F) reiterating that M1 is more effective in improving clearance from cholesterol exposed macrophages. These results supported that chronic exposure to borderline risk cholesterol created a favorable environment for *M.tb* persistence.

**Figure 5:**
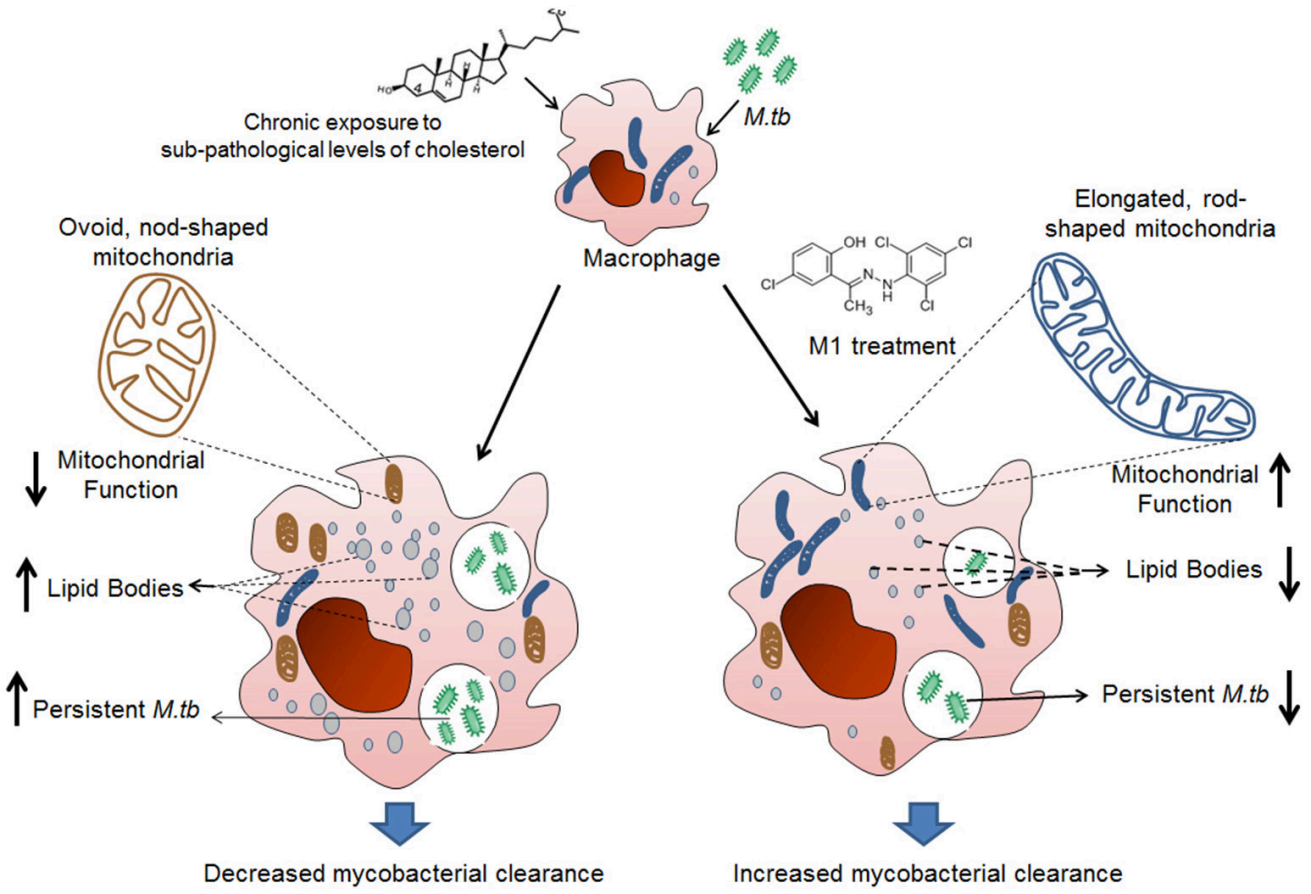
Schematic representation of mechanism involved in M1 mediated restoration of mitochondrial function and anti-mycobacterial activity of THP-1 cells in the pre-disease model mimicking borderline risk cholesterol exposure. Prolonged exposure to sub-pathological levels of cholesterol leads to increase in intracellular lipid bodies, alteration in mitochondrial structure and function that support persistence of *M.tb*. Pharmacological intervention by M1 reversed changes in cholesterol treated infected macrophages, in terms of restoring structure and function of mitochondria and reducing intracellular lipid, thereby increasing the clearance of intracellular mycobacteria.

In summary, we concluded that prolonged exposure to sub-pathological levels of cholesterol leads to increase in intracellular lipid bodies, alteration in mitochondrial structure and function that supported persistence of H37Rv and opportunistic pathogens. Pharmacological intervention by M1 reversed changes in cholesterol treated infected macrophages, thereby increasing the clearance of persistent mycobacteria (Figure 5).

## Discussion

Our study focuses on the role of asymptomatic dyslipidemia as a potential risk factor for tuberculosis progression and the cellular mechanism involved therein. We established a chronic cholesterol model to demonstrate the effect of sub-pathological concentration of cholesterol on the immune response to *M.tb* infection by human macrophages, categorizing the condition as a “pre-disease” state. A “pre-disease” is a designated pre-condition in an asymptomatic individual, identifying which may help to avert major health issues (Viera, 2011). As per global diagnostics standards, serum cholesterol is measured as total cholesterol (TC), low density (LDL), high density (HDL) cholesterol, where a ratio of TC/HDL ranging from 4.5 to 11 is designated as average to moderate risk and a ratio of LDL/HDL cholesterol ranging from 3 to 6 is designated as borderline risk (NABL). Unfortunately, owing to lifestyle changes, much fraction of the population is shifting toward borderline risk. These apparently healthy populations are susceptible to various health risks. TB disease progression is contingent on the physiological background of the host (BoseDasgupta and Pieters, 2014). Over the years, researchers have attempted to identify conditions helping sustained persistence of TB or development of active TB disease from asymptomatic latent cases. While conditions like HIV infections are well-known to increase susceptibility to both fresh *M.tb* infection and activation of latent TB, in this study we, for the first time, showed, that chronic, asymptomatic dyslipidemia in the host can influence the fate of TB persistence and disease progression.

Towards this, we studied immune-competence of macrophages exogenously exposed to sub-pathological levels of cholesterol. The central point of the pre-disease model was a careful titration of cholesterol which will represent the “borderline risk” concentration of cholesterol for macrophages in cultures. We finally found that exposing macrophages to 80 μM of cholesterol for 72 h did not reduce cell viability. This simulated the condition of “borderline risk” cholesterol treatment where cells remained pathologically asymptomatic and viable. The experiments clearly showed that though the cholesterol exposed cells were viable, they failed to exhibit a normal innate immune response, recorded in terms of TNF-α release (Figure 1E) and could not clear the intracellular *M.tb* load efficiently (Figure 1B). Further, we also observed that these cells had dense intracellular lipid bodies upon infection (Figure 3A), exhibiting dysregulated lipid metabolism, giving “foamy” character to these macrophages. Such macrophages were also susceptible to persistent infection by NTM and attenuated mycobacteria, as shown by increased persistence of *M. avium* (Figure 1F) and *M. bovis* BCG (Figure 1G), which are otherwise cleared by untreated macrophages. Deregulation of lipid metabolism in host is known to contribute to the virulence mechanisms of *M.tb*. *M.tb* infection causes perturbations in LXR and PPAR signaling pathways or cholesterol biosynthesis resulting in accumulation of lipid bodies. Several enzymes related to lipid metabolism are up-regulated mediated by PPARγ and LXR transcriptional regulators (Lovewell et al., 2016). The lipid bodies are consumed by the bacteria resulting in their expansion. This enormous requirement of lipid source is mainly because of the lipid-rich cell wall of mycobacteria. Recent studies identified cholesterol as an essential lipid for mycobacterial infection (Larrouy-Maumus, 2015). Additionally, Mce4 transport system of *M.tb* is shown to be essential for cholesterol import into bacterial cells, and genetic perturbation of *mce4* locus results in severe attenuation in a mouse model of *M.tb* infection (D'Avila et al., 2006; Pandey and Sassetti, 2008; Almeida et al., 2009; Lee et al., 2013).

Some of the earlier experiments have established the role of cholesterol in *M.tb* entry. They noted cholesterol specific sensors present on the cholesterol-rich membrane domains of the host cells (Kaul et al., 2004) and accumulation of cholesterol at the site of bacterial entry (Gatfield and Pieters, 2000). Further, Viswanathan et al. showed that brief exposure to very high cholesterol treatment (1 mM) facilitated *M.tb* entry through these lipid rafts in macrophage cell lines (Viswanathan et al., 2015). In contrast to these studies, we used much less concentration of cholesterol with a prolonged exposure time in our model. This was primarily keeping the objective of understanding the role of chronic exposure of sub-pathological cholesterol on *M.tb* infection. We confirmed that our conditions though did not support *M.tb* entry, assisted increased persistence of intracellular mycobacteria, besides making the cholesterol exposed macrophages susceptible to persistent infection by opportunistic mycobacteria. This could be linked to impaired immune function, dysregulated lipid accumulation and mitochondrial dysfunction in cholesterol treated macrophages. Treatment of these cells with a small molecule M1 reinstated the ability to clear intracellular mycobacteria in these macrophages by restoring mitochondrial structure and function besides reducing intracellular lipid bodies (Figure 5).

Moderate levels of cholesterol lead to mitochondrial dysfunction by causing structural and functional aberrations (Asalla et al., 2016). Mitochondria are poorly constituted with cholesterol in their lipid bilayers as compared to that of the plasma membrane. However, the low level of cholesterol in the mitochondrial inner membrane (MIM) favors important physiological functions including synthesis of bile acids in hepatocytes, maintenance of intact mitochondrial membrane structure, homeostatic membrane potential, OXPHOS and so on. In pathological conditions, accumulated cholesterol in MIM alter not only the membrane organization but also mitochondria-associated immune cell functions including infection mediated apoptosis and necrosis (Garcia-Ruiz et al., 2009). There is clinical evidence indicating familial hypercholesteremia as a risk factor for *M.tb* infection. Supporting the notion, administration of cholesterol-reducing drugs, such as statins is reported to control mycobacterial burden (Parihar et al., 2014). Not only mycobacteria, but treatment with lovastatin and atorvastatin reduced the growth of *Salmonella enterica* in murine macrophage cell line and *in vivo* mouse model of infection, respectively (Catron et al., 2004). Treatment with simvastatin significantly reduced the growth of *Chlamydia pneumonia* in mice (Erkkila et al., 2005).

Mitochondrial function is closely linked both to mitochondrial morphology and distribution, alterations in which reflect shifts in cell activity (Escobar-Henriques and Langer, 2006). Normal mitochondrial morphology is represented by networks of elongated rod-shaped mitochondria, whereas solitary, spherical or nod-shape has been linked to abnormal mitochondrial physiology in several pathological conditions (Cataldo et al., 2010). In our experiments, we found that cholesterol treated THP-1 cells had fragmented, ovoid and nod-shaped mitochondria as observed by confocal and TEM microscopy, which was more pronounced upon infection (Figures 2A,B). Heterogeneity in the mitochondrial forms was found in all conditions owing to dynamicity of fission and fusion processes. TEM images also showed the accumulated lipid bodies in cholesterol-treated cells. We experienced difficulties in staining these cells due to the presence of these lipid bodies. As shown in earlier studies (Fine-Coulson et al., 2015), we also observe that *M.tb* infection alone affected mitochondrial morphology as compared to uninfected cells (Figures 2A,B), however, mitochondria in cholesterol treated infected cells were distinctly solitary, swollen, and spherical in shape (Figure 2B lower panel, column ii) representing extreme stress that may lead to mitochondrial dysfunction.

We next correlated the alteration in mitochondrial morphology with expression of the related genes. The shape and structure of mitochondria are maintained by two opposing forces: fission and fusion (Reddy, 2009), the imbalance between the same leads to abnormal mitochondrial dynamics. Fusion and fission are controlled by evolutionarily conserved, large GTPases belonging to the family of dynamin-like MFN-1, MFN-2, OPA-1, DRP-1, and MFF. Alterations in the homeostasis affect mitochondrial morphology and thus function. While mitochondrial fission is mediated by *drp-1* and *mff*, fusion is mediated by *mfn-1, mfn-2*, and *opa-1*. We, therefore, investigated the expression status of the structural genes *mfn*-1 and *mfn*-2, *drp*-1, and *mff* by qRT-PCR. In our experiments, we observed that both *mfn-1* and *mfn-2* were downregulated in cholesterol treated infected cells. The reduced levels of either *mfn-1* or *mfn-2* cause altered mitochondrial fusion resulting in a change in mitochondrial morphology (rod-shaped mitochondria turn into nod shaped). It was interesting to note that sub-pathological cholesterol treatment alone reduced the expression of the mitochondrial fusion gene, *mfn-2* by 47.18% (Figure 2D) and increased the expression of fission gene *drp-1* by ~20% (Figure 2E), paving the way to mitochondrial fragmentation and therefore nod shaped structures.

Mitochondrial functional parameters are closely related to its structure, to the extent that changes in the mitochondrial reticulum due mutations of mitochondrial or nuclear-encoded genes are linked to mitochondrial oxidative phosphorylation (Zanna et al., 2008). Further, mitochondrial membranes may be coupled electrically when networked and membrane potential of individual mitochondria may change inter-dependently. We observed that MMP was reduced upon *M.tb* infection irrespective of cholesterol treatment (Figure 2G) indicating that infection and not cholesterol is responsible for altered MMP. In addition to changes in MMP, modifications in mitochondrial shape and distribution may be concomitant with anomalous oxidative phosphorylation and production of ATP. Testing for this possibility, we compared ATP/ADP ratios in these categories of infected cells. Like MMP, ATP/ADP, ratios were reduced equally during infection of cholesterol treated and untreated cells (Figure 2H), suggesting abnormality in ATP production as a function of infection rather than cholesterol treatment.

These changes in mitochondrial morphology, expression of the genes regulating the same and mitochondrial function could be reversed by the intervention with M1 that is known to restore mitochondrial structure (Wang et al., 2012; Figures 2). The hydrazone moiety small molecule M1 was identified by Wang et al., where it was shown through mutants (mfn-1/2 or opa-1 KOs) that M1 restores mitochondrial morphology in cells with a basal mitochondrial fusion defect. However, we observed that M1 restored levels of fission gene *mff* in cholesterol-treated cells. The reason behind the partial restoration of fission genes in M1 treated cells is presently unclear.

With *M.tb* infection prominently affecting both mitochondrial fission and fusion genes in cholesterol treated THP-1 cells, the balance of which can be restored by M1, it would be interesting to investigate the mode of action of M1 like molecule in restoring mitochondrial activity in cholesterol exposed cells. Further, M1 treatment substantially restored the mitochondrial morphology in both treated and untreated infected cells, though TEM images clearly indicated that extent of restoration of mitochondrial morphology was more distinct in cholesterol treated infected cells (Figures 2A,B). In terms of restoration of mitochondrial function, M1 treatment could restore MMP equally in untreated and treated infected cells (Figure 2G) but could restore ATP/ADP ratio only in cholesterol treated infected cells (Figure 2H). We also observed that M1 treatment reduced accumulation of intracellular lipid bodies, specifically in cholesterol treated infected cells. Additionally, M1 treatment could also augment clearance of intracellular persistent *M.tb* more efficiently in cholesterol treated infected cells as compared to untreated infected cells (Figure 3D).

We further supported our inferences from cell culture experiments by infection assays in PBMC-derived macrophages from clinically healthy volunteers. The subjects recruited were analyzed for total serum cholesterol (TC), LDL, and HDL cholesterol and were categorized based on TC/HDL and LDL/HDL ratios. The categorization followed the global diagnostics standards as discussed earlier where those with TC/HDL ratio falling between 4.5–11 and LDL/HDL ratio in the range 3–6 were categorized as BLC, while below this range were considered NC. We observed similar impact of 80 μM treatment of cholesterol in both THP-1 model and PBMC derived macrophages from NC confirming the reproducibility of cell culture studies in primary cells (Figure 4A). We also observed that PBMC-derived macrophages from BLC were inefficient in clearing H37Rv as compared to that by NC (Figure 4B). Interestingly, as observed in THP-1 model, M1 treatment cleared H37Rv from cholesterol treated PBMC derived macrophages from BLC more effectively than untreated (Figure 4F), corroborating the observation that M1 treatment enhanced clearance of intracellular *M.tb* more efficiently in cholesterol exposed infected cells (Figure 3D).

TB can be prevented, and one step toward it is to protect the susceptible population. Several risk factors that have been identified which contribute to TB susceptibility and persistence including HIV infection, malnutrition, diabetes, cancer, smoking and so on (CDC, 2016). Of late, the role of lysosomal storage disorders as a susceptible factor for TB progression is being investigated. The mutants of lysosomal cathepsins incapacitate the lysosomes in mycobacterial clearance (Berg et al., 2016) and thereby promote mycobacterial persistence. It would also be worthwhile to investigate the role of lysosomal storage disorder upon exposure to borderline risk cholesterol and its impact on tuberculosis infection. Through this study, we establish that individuals with borderline risk cholesterol profiles, though clinically healthy, but are at the risk of susceptibility to persistent infections like that of TB, as they are not able to respond adequately. In this study, we could show that these seemingly harmless levels of cholesterol could convert normal macrophages into “foamy” macrophages, caused asymptomatic damage to host cell mitochondria, which becomes apparent when *M.tb* infection occurred, in terms of both mitochondrial structure and function. With these perfectly viable cells failing to clear the intracellular mycobacterial load and developing susceptibility to persistent infections by non-tuberculous and attenuated mycobacteria, we propose that borderline risk cholesterol profile can be one of the factors leading to susceptibility to *M.tb* persistence. Systematic population studies can be designed towards validating these baseline laboratory observations to consider it as a “pre-disease” screening criterion. With the supplementation of small molecule M1 that restored mitochondrial structural and functional integrity along with increasing *M.tb* clearance, more efficiently in cholesterol exposed macrophages, we propose exploring such molecules as yet another strategy for host-directed therapy in TB disease management.

## Author contributions

SA and SB conceived and designed the study; SA performed the experiments; SA, KM, and SB analyzed the data and wrote the manuscript. All authors have read and approved the final manuscript.

### Conflict of interest statement

The authors declare that the research was conducted in the absence of any commercial or financial relationships that could be construed as a potential conflict of interest.

## Footnotes

^1^NABL (National Accreditation Board for Testing and Calibration Laboratories).http://www.nabl-india.org/
