# Supplementary material for: Small Molecule Mediated Restoration of Mitochondrial Function Augments Anti-Mycobacterial Activity of Human Macrophages Subjected to Cholesterol Induced Asymptomatic Dyslipidemia
Source: Front Cell Infect Microbiol. 2017 Oct 10;7:439. doi: 10.3389/fcimb.2017.00439 (PMC5641336; doi:10.3389/fcimb.2017.00439)
Supplement: Supplementary file 1 [file Presentation1.ppt]

## Slide 1
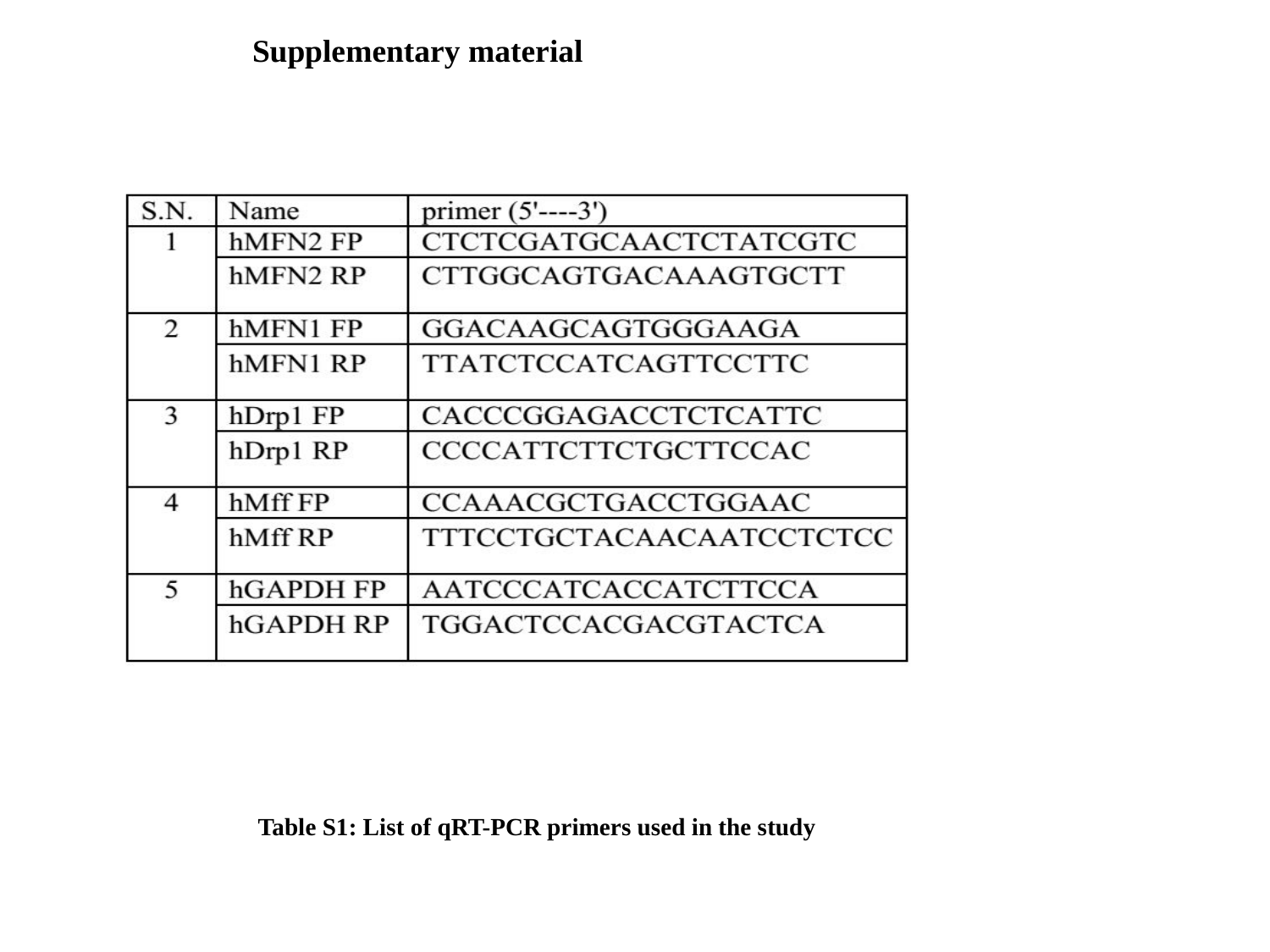

Supplementary material
Table S1: List of qRT-PCR primers used in the study

## Slide 2
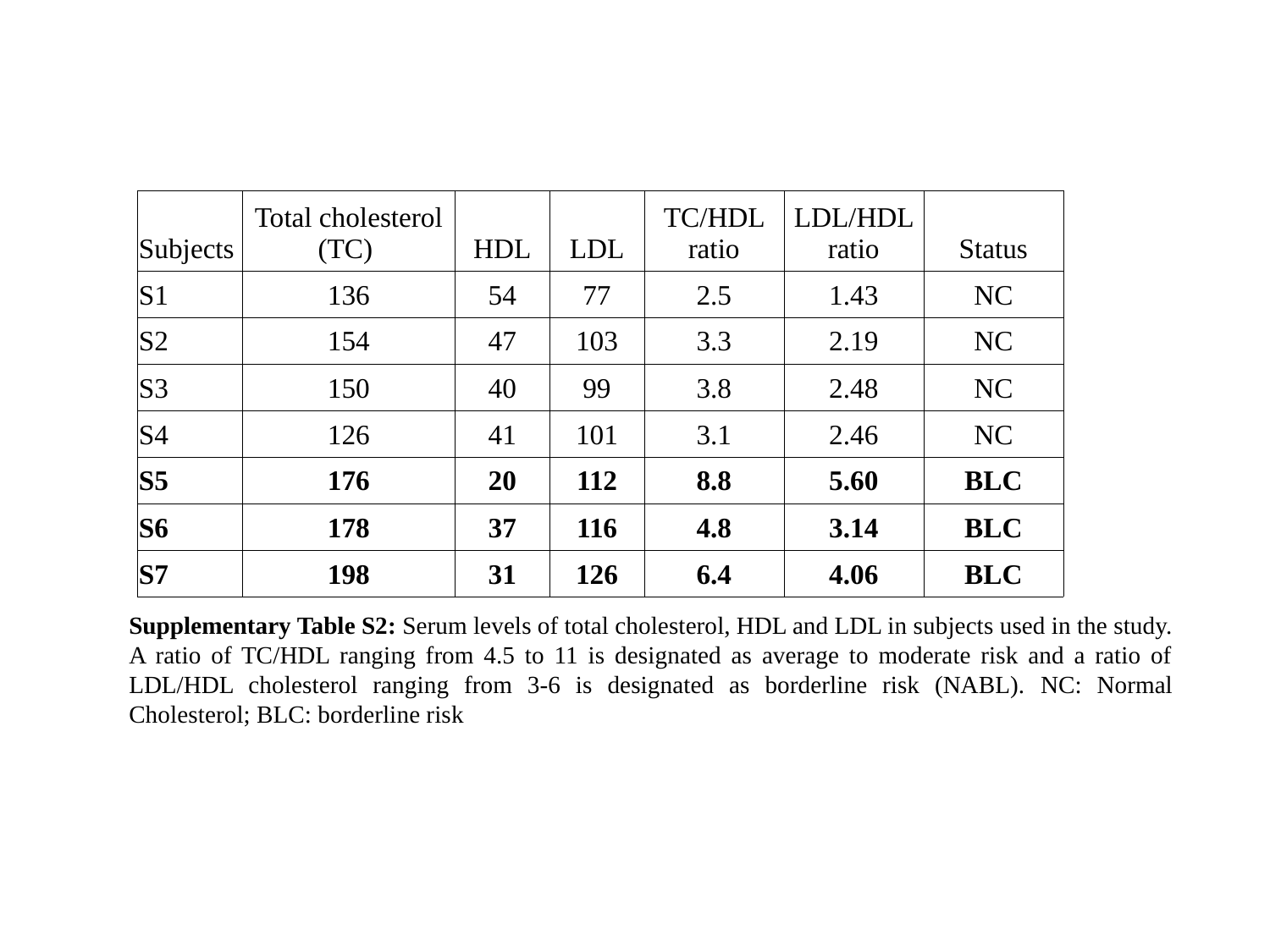

| Subjects | Total cholesterol (TC) | HDL | LDL | TC/HDL ratio | LDL/HDL ratio | Status |
| --- | --- | --- | --- | --- | --- | --- |
| S1 | 136 | 54 | 77 | 2.5 | 1.43 | NC |
| S2 | 154 | 47 | 103 | 3.3 | 2.19 | NC |
| S3 | 150 | 40 | 99 | 3.8 | 2.48 | NC |
| S4 | 126 | 41 | 101 | 3.1 | 2.46 | NC |
| S5 | 176 | 20 | 112 | 8.8 | 5.60 | BLC |
| S6 | 178 | 37 | 116 | 4.8 | 3.14 | BLC |
| S7 | 198 | 31 | 126 | 6.4 | 4.06 | BLC |
Supplementary Table S2: Serum levels of total cholesterol, HDL and LDL in subjects used in the study. A ratio of TC/HDL ranging from 4.5 to 11 is designated as average to moderate risk and a ratio of LDL/HDL cholesterol ranging from 3-6 is designated as borderline risk (NABL). NC: Normal Cholesterol; BLC: borderline risk

## Slide 3
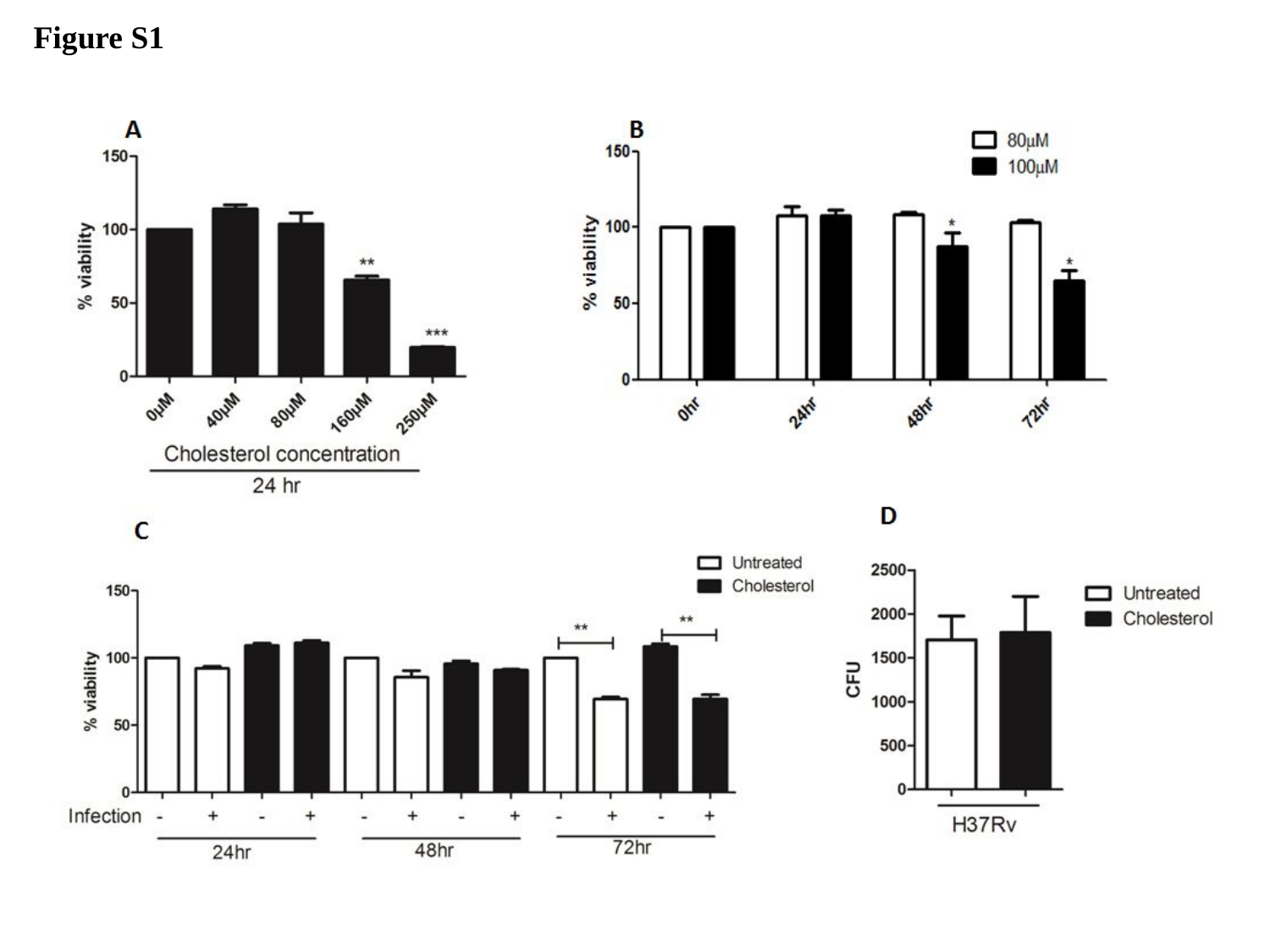

Figure S1

## Slide 4
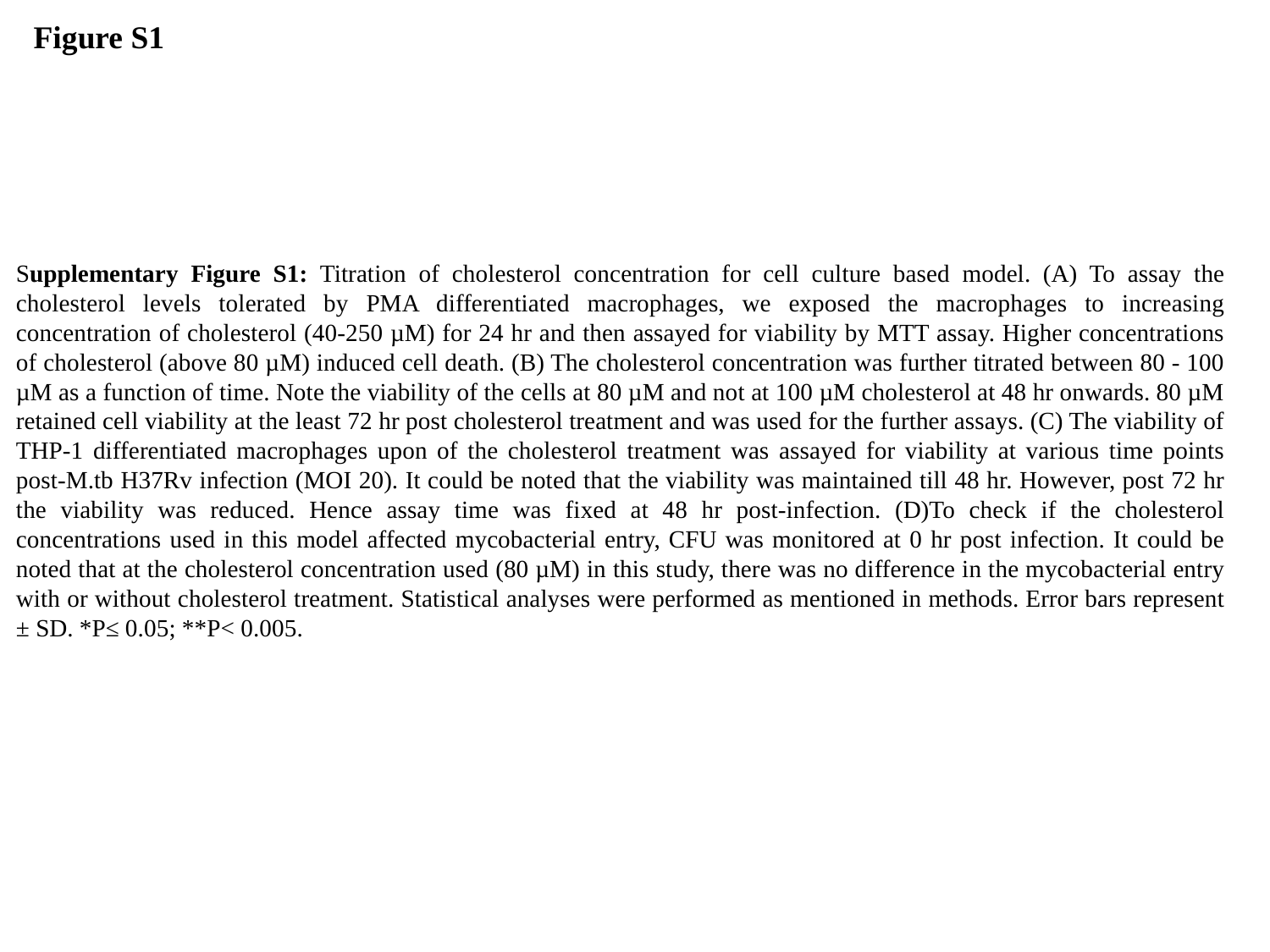

Figure S1
Supplementary Figure S1: Titration of cholesterol concentration for cell culture based model. (A) To assay the cholesterol levels tolerated by PMA differentiated macrophages, we exposed the macrophages to increasing concentration of cholesterol (40-250 µM) for 24 hr and then assayed for viability by MTT assay. Higher concentrations of cholesterol (above 80 µM) induced cell death. (B) The cholesterol concentration was further titrated between 80 - 100 µM as a function of time. Note the viability of the cells at 80 µM and not at 100 µM cholesterol at 48 hr onwards. 80 µM retained cell viability at the least 72 hr post cholesterol treatment and was used for the further assays. (C) The viability of THP-1 differentiated macrophages upon of the cholesterol treatment was assayed for viability at various time points post-M.tb H37Rv infection (MOI 20). It could be noted that the viability was maintained till 48 hr. However, post 72 hr the viability was reduced. Hence assay time was fixed at 48 hr post-infection. (D)To check if the cholesterol concentrations used in this model affected mycobacterial entry, CFU was monitored at 0 hr post infection. It could be noted that at the cholesterol concentration used (80 µM) in this study, there was no difference in the mycobacterial entry with or without cholesterol treatment. Statistical analyses were performed as mentioned in methods. Error bars represent ± SD. *P≤ 0.05; **P< 0.005.

## Slide 5
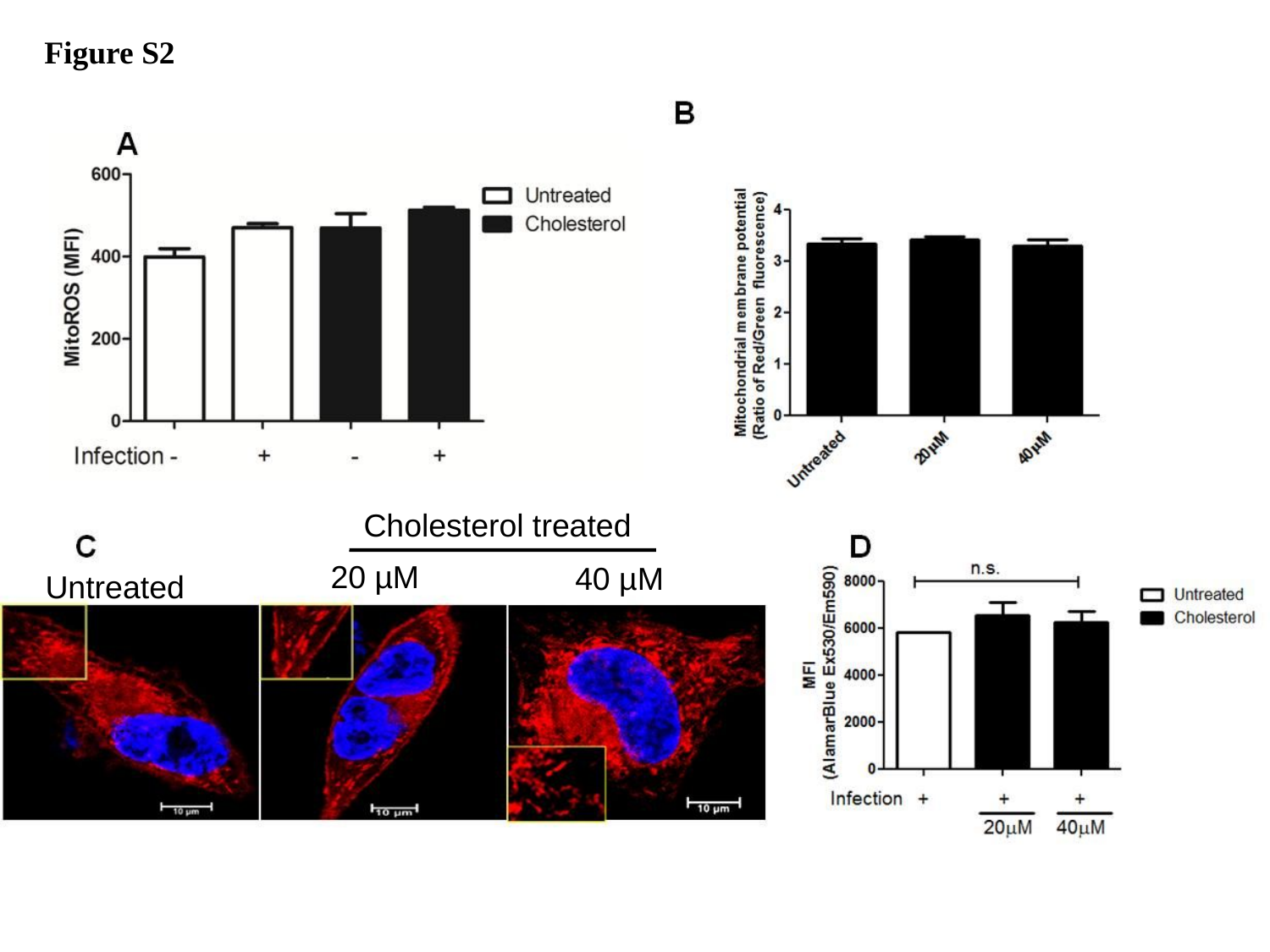

Figure S2
Cholesterol treated
20 µM
40 µM
Untreated

## Slide 6
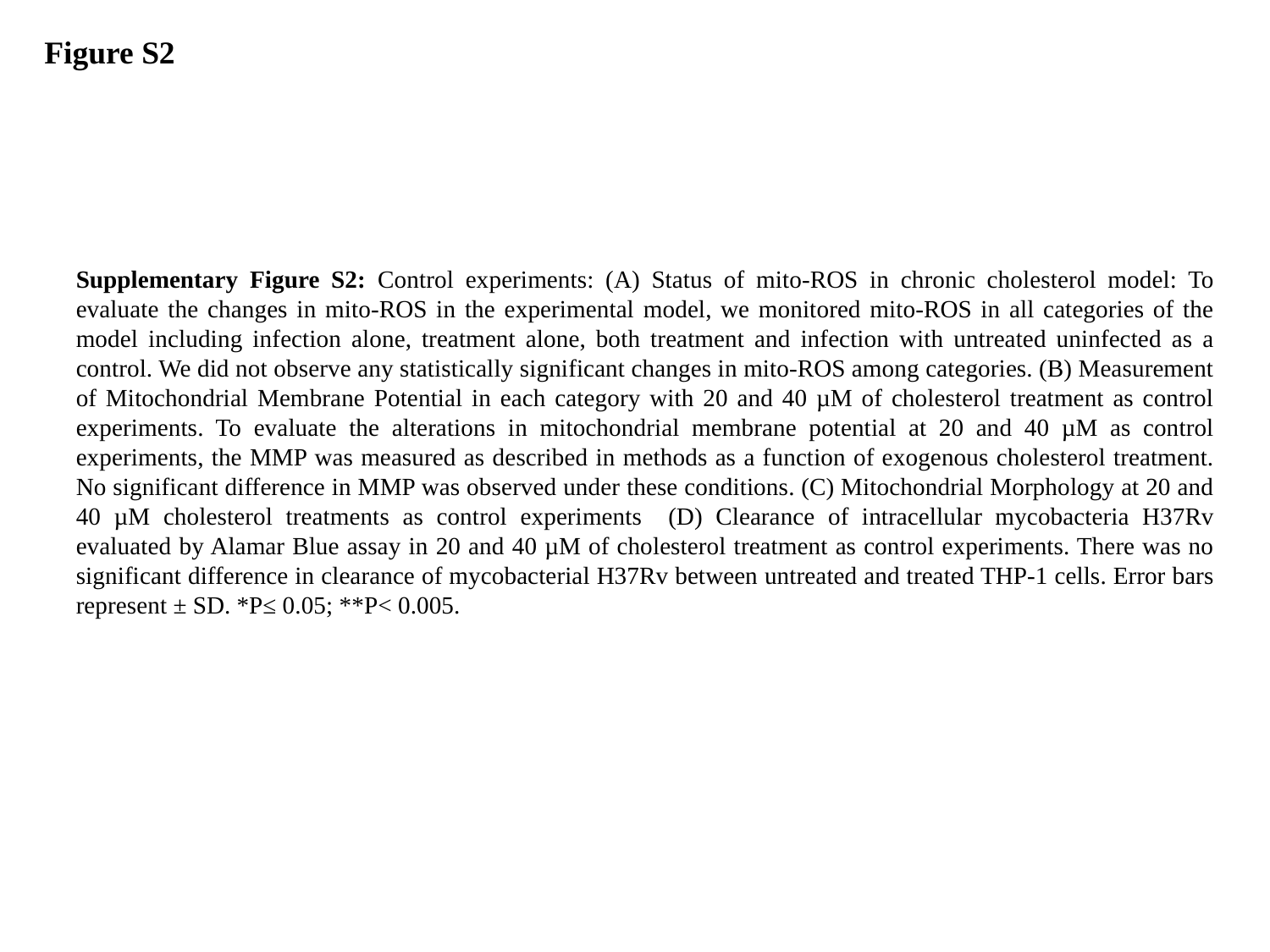

Figure S2
Supplementary Figure S2: Control experiments: (A) Status of mito-ROS in chronic cholesterol model: To evaluate the changes in mito-ROS in the experimental model, we monitored mito-ROS in all categories of the model including infection alone, treatment alone, both treatment and infection with untreated uninfected as a control. We did not observe any statistically significant changes in mito-ROS among categories. (B) Measurement of Mitochondrial Membrane Potential in each category with 20 and 40 µM of cholesterol treatment as control experiments. To evaluate the alterations in mitochondrial membrane potential at 20 and 40 µM as control experiments, the MMP was measured as described in methods as a function of exogenous cholesterol treatment. No significant difference in MMP was observed under these conditions. (C) Mitochondrial Morphology at 20 and 40 µM cholesterol treatments as control experiments (D) Clearance of intracellular mycobacteria H37Rv evaluated by Alamar Blue assay in 20 and 40 µM of cholesterol treatment as control experiments. There was no significant difference in clearance of mycobacterial H37Rv between untreated and treated THP-1 cells. Error bars represent ± SD. *P≤ 0.05; **P< 0.005.

## Slide 7
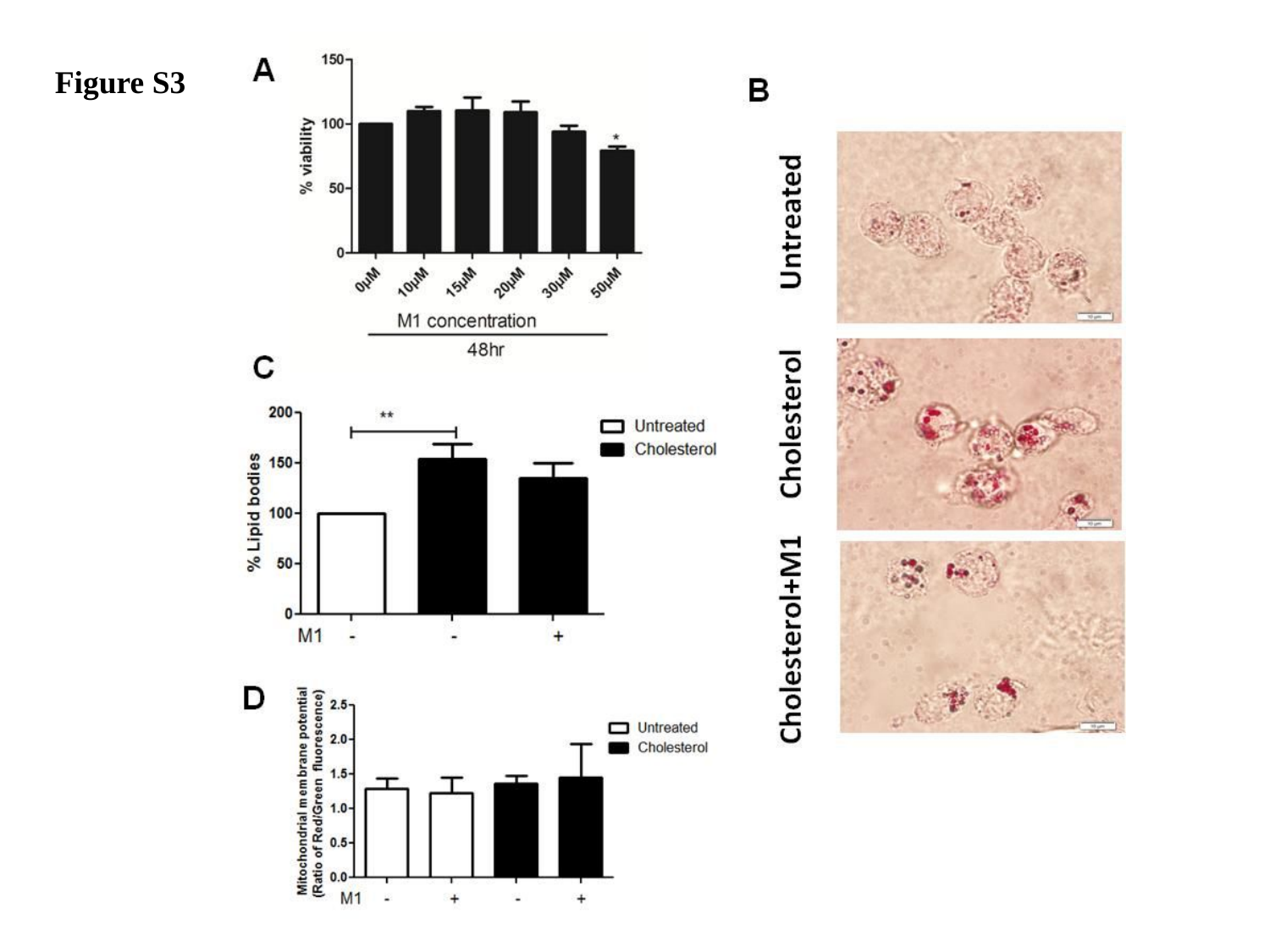

Figure S3

## Slide 8
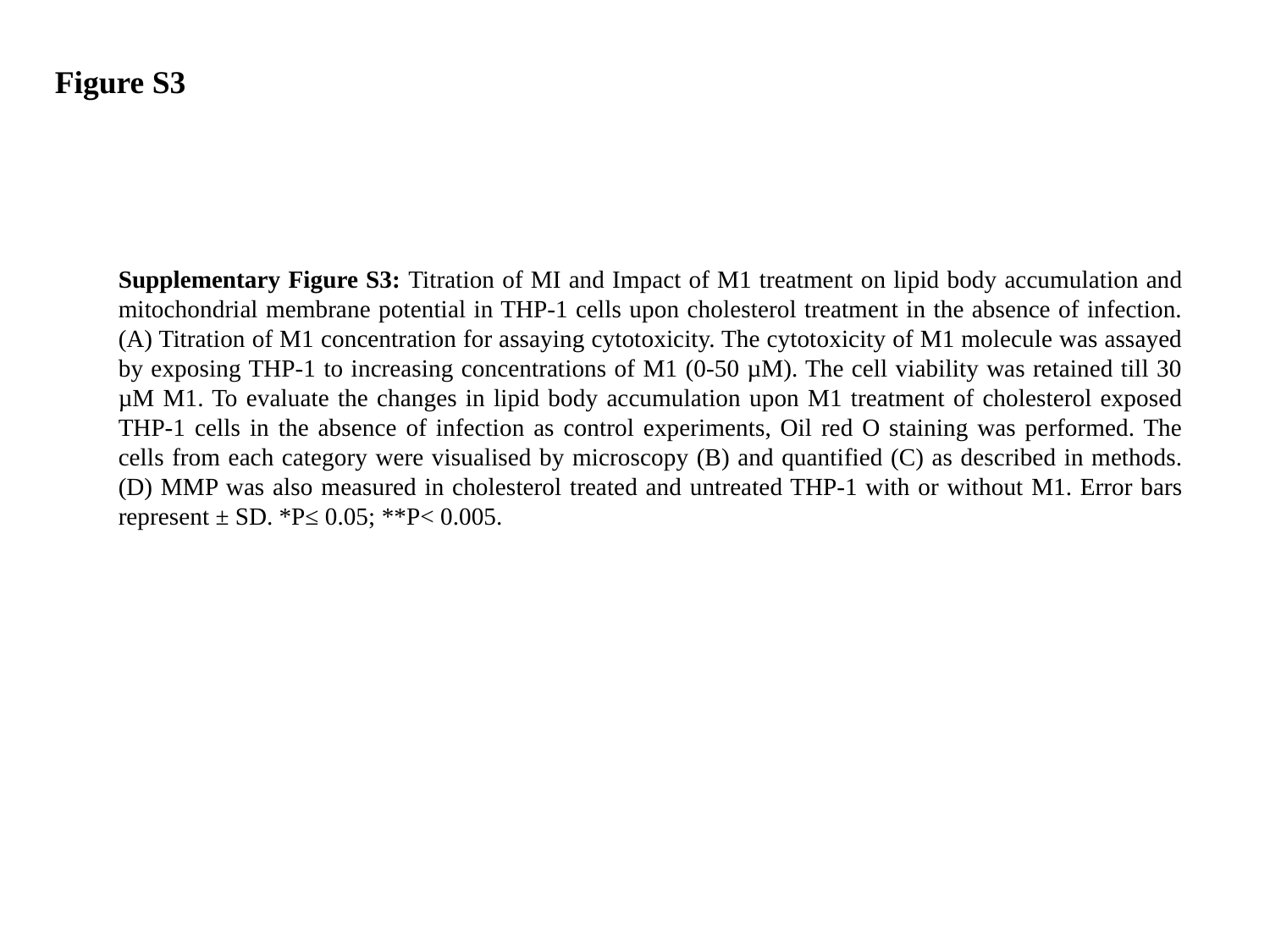

Figure S3
Supplementary Figure S3: Titration of MI and Impact of M1 treatment on lipid body accumulation and mitochondrial membrane potential in THP-1 cells upon cholesterol treatment in the absence of infection. (A) Titration of M1 concentration for assaying cytotoxicity. The cytotoxicity of M1 molecule was assayed by exposing THP-1 to increasing concentrations of M1 (0-50 µM). The cell viability was retained till 30 µM M1. To evaluate the changes in lipid body accumulation upon M1 treatment of cholesterol exposed THP-1 cells in the absence of infection as control experiments, Oil red O staining was performed. The cells from each category were visualised by microscopy (B) and quantified (C) as described in methods. (D) MMP was also measured in cholesterol treated and untreated THP-1 with or without M1. Error bars represent ± SD. *P≤ 0.05; **P< 0.005.

## Slide 9
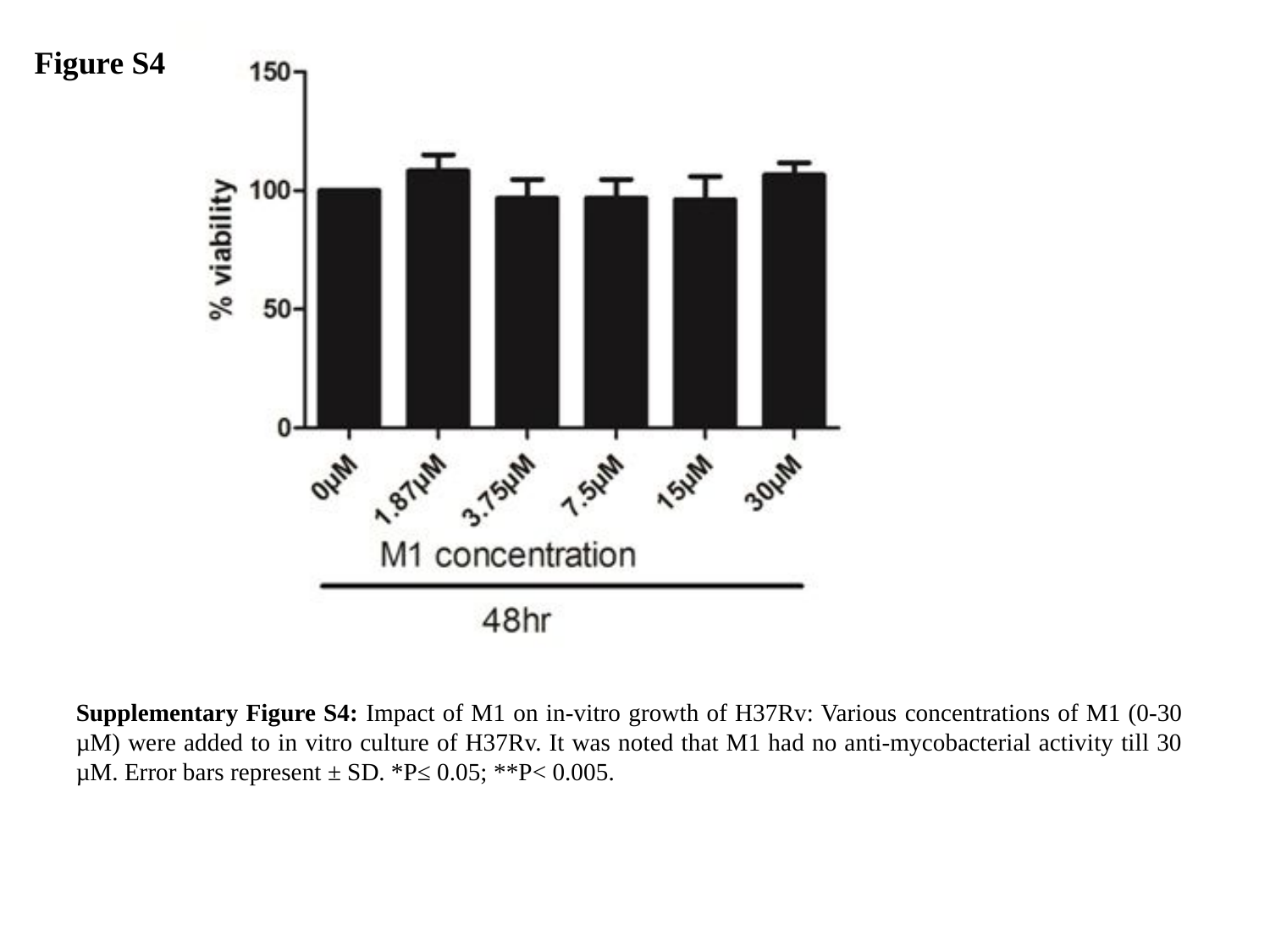

Figure S4
Supplementary Figure S4: Impact of M1 on in-vitro growth of H37Rv: Various concentrations of M1 (0-30 µM) were added to in vitro culture of H37Rv. It was noted that M1 had no anti-mycobacterial activity till 30 µM. Error bars represent ± SD. *P≤ 0.05; **P< 0.005.

## Slide 10
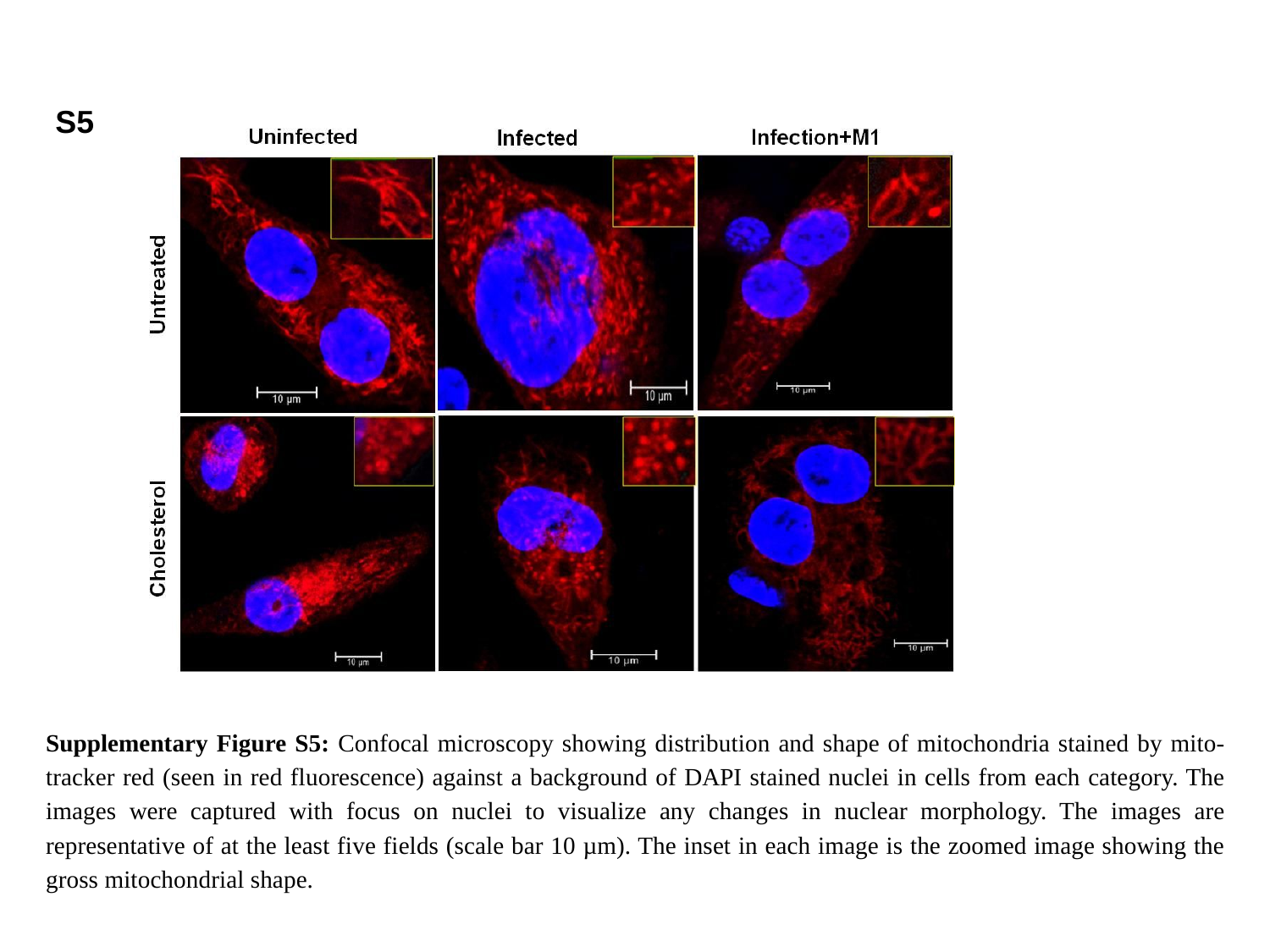

S5
Supplementary Figure S5: Confocal microscopy showing distribution and shape of mitochondria stained by mito-tracker red (seen in red fluorescence) against a background of DAPI stained nuclei in cells from each category. The images were captured with focus on nuclei to visualize any changes in nuclear morphology. The images are representative of at the least five fields (scale bar 10 µm). The inset in each image is the zoomed image showing the gross mitochondrial shape.
